# Minor Type IV Collagen α5 Chain Promotes Cancer Progression through Discoidin Domain Receptor-1

**DOI:** 10.1371/journal.pgen.1005249

**Published:** 2015-05-19

**Authors:** Qian Xiao, Yan Jiang, Qingbo Liu, Jiao Yue, Chunying Liu, Xiaotong Zhao, Yuemei Qiao, Hongbin Ji, Jianfeng Chen, Gaoxiang Ge

**Affiliations:** 1 Key Laboratory of Systems Biology, Innovation Center for Cell Signaling Network, Institute of Biochemistry and Cell Biology, Shanghai Institutes for Biological Sciences, Chinese Academy of Sciences, Shanghai, People’s Republic of China; 2 State Key Laboratory of Cell Biology, Institute of Biochemistry and Cell Biology, Shanghai Institutes for Biological Sciences, Chinese Academy of Sciences, Shanghai, People’s Republic of China; Dana Farber Cancer Institute, UNITED STATES

## Abstract

Type IV collagens (Col IV), components of basement membrane, are essential in the maintenance of tissue integrity and proper function. Alteration of Col IV is related to developmental defects and diseases, including cancer. Col IV α chains form α1α1α2, α3α4α5 and α5α5α6 protomers that further form collagen networks. Despite knowledge on the functions of major Col IV (α1α1α2), little is known whether minor Col IV (α3α4α5 and α5α5α6) plays a role in cancer. It also remains to be elucidated whether major and minor Col IV are functionally redundant. We show that minor Col IV α5 chain is indispensable in cancer development by using α5(IV)-deficient mouse model. Ablation of α5(IV) significantly impeded the development of Kras^G12D^-driven lung cancer without affecting major Col IV expression. Epithelial α5(IV) supports cancer cell proliferation, while endothelial α5(IV) is essential for efficient tumor angiogenesis. α5(IV), but not α1(IV), ablation impaired expression of non-integrin collagen receptor discoidin domain receptor-1 (DDR1) and downstream ERK activation in lung cancer cells and endothelial cells. Knockdown of DDR1 in lung cancer cells and endothelial cells phenocopied the cells deficient of α5(IV). Constitutively active DDR1 or MEK1 rescued the defects of α5(IV)-ablated cells. Thus, minor Col IV α5(IV) chain supports lung cancer progression via DDR1-mediated cancer cell autonomous and non-autonomous mechanisms. Minor Col IV can not be functionally compensated by abundant major Col IV.

## Author Summary

Collagens, the major extracellular matrix components in most vertebrate tissues, provide cells with structural and functional support. Collagens are trimers of collagen α chains. Multiple trimers are formed by highly homologous α chains for certain types of collagens (e.g. α1α1α2, α3α4α5 and α5α5α6 heterotrimers for type IV collagen). Type IV collagens are named as major type (α1α1α2) or minor type (α3α4α5 and α5α5α6), mainly reflecting the abundance and tissue distribution, but not the importance of their biological functions. High similarity in sequence and domain structure of the α chains does not necessarily imply that major and minor type IV collagens share the same cell surface receptors and intracellular signaling pathways. In this study, we generated an α5(IV) chain deficient mouse model lacking minor type IV collagens. We found that the mutant mice have delayed development of Kras^G12D^-driven lung cancer without affecting major type IV collagen expression. α5(IV), but not α1(IV), ablation impaired non-integrin collagen receptor discoidin domain receptor-1 (DDR1)-ERK signaling, suggesting that major and minor type IV collagens are functionally distinct from each other.

## Introduction

Basement membranes (BMs), specialized extracellular matrices separating epithelial and endothelial cells from underlying mesenchyme, provide cells with structural support, as well as morphogenic and functional cues [1–3]. Type IV collagens (Col IV) are major components of BMs [1,3]. Three triple helical protomers, α1α1α2, α3α4α5 and α5α5α6, are formed by the Col IV α chains that further form collagen networks [4,5]. α1α1α2, the major Col IV, is widely expressed as a component of all BMs. α3α4α5 and α5α5α6, known as minor Col IV, have much restricted tissue distribution [4,5].

Col IV-initiated signals are essential survival and growth cues for liver metastasis in diverse tumor types [6]. BM proteins produced by mouse Engelbrecht Holm-Swarm sarcoma, known as Matrigel, enhanced the tumorigenicity of human cancer cells [7]. BM proteins, including α1(IV), protect small cell lung cancer cells from chemotherapy-induced apoptosis [8]. Angiogenesis, required by tumors to supply nutrients and oxygen, and to evacuate metabolic wastes, is dependent on correct interaction between endothelial cells and the vascular BMs [1,9,10]. Col IV plays crucial roles in supporting endothelial cell proliferation and migration. Blood vessel formation and survival are connected with proper collagen synthesis and deposition in BMs. Col IV, by binding to cell surface receptors, activates intracellular signaling events to promote cell survival, proliferation and tumorigenesis [5]. Loss of integrin α1β1 ameliorates Kras^G12D^-induced lung cancer [11,12]. β1 integrin and its downstream effecter focal adhesion kinase (FAK) are critical in mediating resistance to anoikis, chemotherapy-induced cell death and metastasis [6,8,11].

Despite Col IV is extensively studied, majority of the works focused on the functions of major Col IV, or unfortunately did not distinguish the roles of major and minor Col IV. It is largely unknown whether minor Col IV plays a role in cancer development. It also remains to be elucidated whether major and minor Col IV signal through the same cell surface receptors and intracellular signaling pathways and whether they can functionally compensate for each other.

In the present study, we demonstrate that minor Col IV α5 chain is indispensable in lung cancer development by using α5(IV)-deficient mouse model. α5(IV) supports lung cancer progression via cancer cell autonomous and non-autonomous mechanisms. α5(IV), but not α1(IV), promotes lung cancer cell proliferation and tumor angiogenesis through non-integrin collagen receptor DDR1-mediated ERK activation. The functions of minor Col IV can not be compensated by abundant major Col IV.

## Results

### α5(IV) chain is required for lung cancer progression

A *LacZ* gene trap cassette including *En2* splice acceptor/*ECMV IRES*/*LacZ*/*SV40* polyadenylation site was inserted into intron 35 of mouse *Col4a5* gene on chromosome X to generate *Col4a5* knockout mice (S1A and S1B Fig) [13]. RT-PCR analyses demonstrated the absence of *Col4a5* mRNA in the KO tissues (S1C and S1D Fig). The *LacZ* reporter reflects endogenous *Col4a5* expression. Strong *LacZ* staining was observed in lung bronchia (S1E Fig). Immunofluorescent staining demonstrated that α5(IV) chain is expressed in lung bronchia at high levels, and in lung alveolar epithelial cells at lower levels in *Col4a5* ^+/Y^ (hereafter refereed as wild-type, WT) mice (S1F Fig). The α5(IV) signal is absent in *Col4a5* ^*LacZ*/Y^ (hereafter refereed as knockout, KO) lungs (S1F Fig), further demonstrating that the mutant *Col4a5* allele is indeed null.

**Fig 1:**
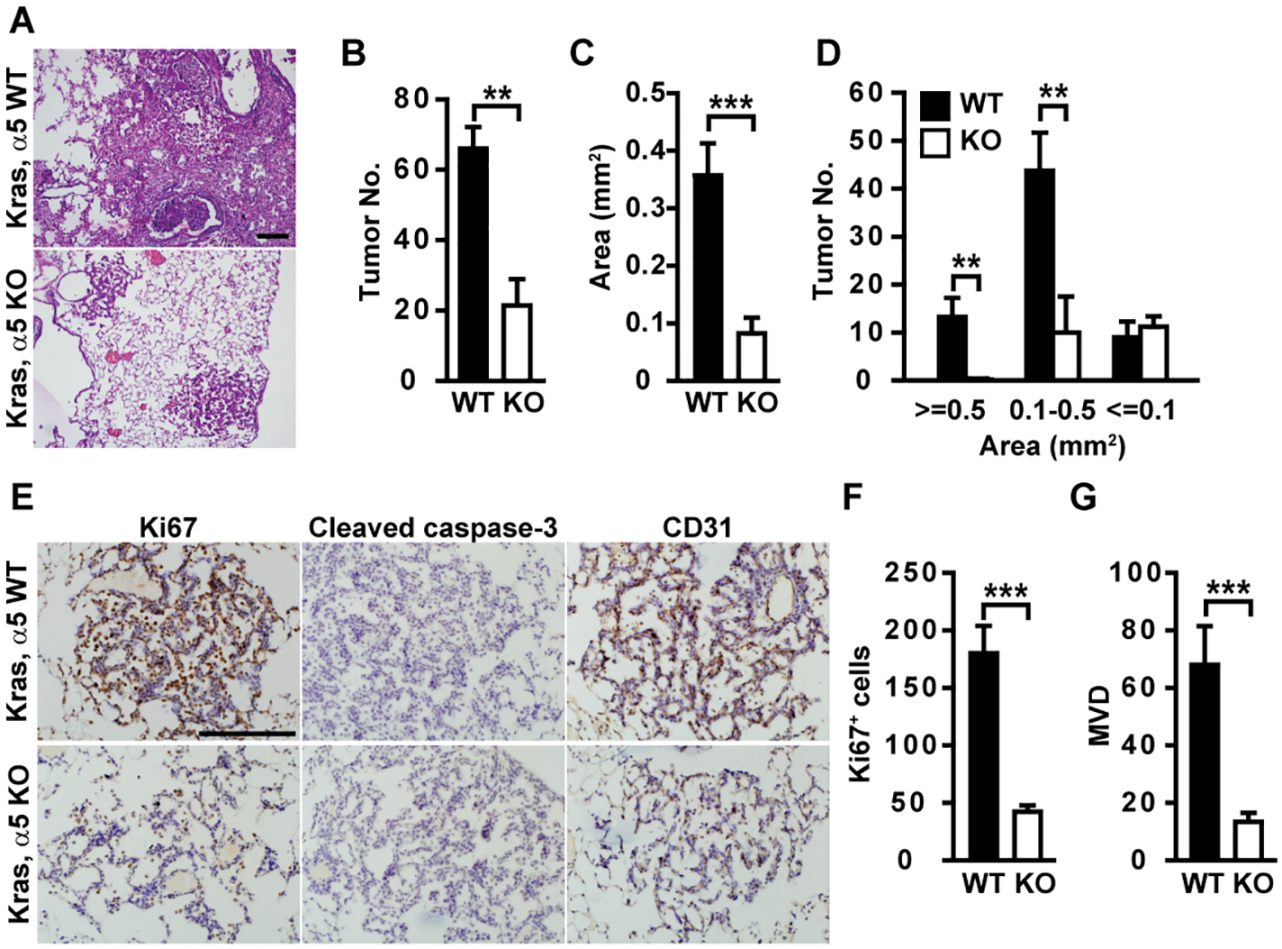
Tumor development is delayed in α5(IV)-deficient mice. (A) H&E staining of lungs of Kras/α5 WT and Kras/α5 KO mice. Arrows indicate hemorrhage lesions. (B-D) Quantification of tumor volume and numbers in H&E-stained lung sections from Kras/α5 WT (n = 6) and Kras/α5 KO (n = 6) mice. (E) Lung tumor sections were stained with anti-Ki67, anti-cleaved caspase-3, or anti-CD31 antibodies. No apoptotic signal was evident in both groups. (F and G) Quantitative proliferative indices (F) and microvascular density (MVD) (G) were measured. Data are presented as mean ± SEM. **P < 0.01, ***P < 0.001. Scale bars: 200μm.

Oncogenic Kras^G12D^ drives lung cancer onset and progression. In contrast to large, multifocal tumors formed in *Kras* ^G12D^; *Col4a5* ^*+*/Y^ (Kras/α5 WT) mice, significantly less tumors developed in *Kras* ^G12D^; *Col4a5* ^*LacZ*/Y^ (Kras/α5 KO) mice (Fig 1A and 1B). Tumors in Kras/α5 KO mice were significantly smaller than those in Kras/α5 WT mice (Fig 1A and 1C). α5(IV) ablation dramatically reduced the number of large tumors (>0.5 mm^2^), but had no profound effect on the number of small tumors (<0.1 mm^2^) (Fig 1D), indicating that α5(IV) is mainly involved in regulating tumor progression, but not tumor onset.

BM proteins promote cancer cell proliferation and protect cancer cells from apoptosis. Tumors in Kras/α5 KO mice had significantly reduced tumor cell proliferation (Fig 1E and 1F), compared with those in Kras/α5 WT mice. Few apoptotic signal was evident in both groups (Fig 1E). Hemorrhage was evident in α5 KO lungs, but not in WT lungs (Fig 1A). Hemorrhage lesions indicate improper organization of capillaries and blood vessels in α5 KO lungs. As tumor angiogenesis provides tumor cells nutrients and oxygen necessary for sustained tumor growth, this promoted us to examine whether neo-angiogenesis was compromised in Kras/α5 KO tumors. Indeed, tumors in Kras/α5 KO mice were significantly less vascularized (Fig 1E and 1G). Thus, reduction in tumor cell proliferation and tumor angiogenesis account for delayed tumor progression in Kras/α5 KO mice.

### Epithelial α5(IV) supports tumor cell growth and tumorigenicity

**Fig 2:**
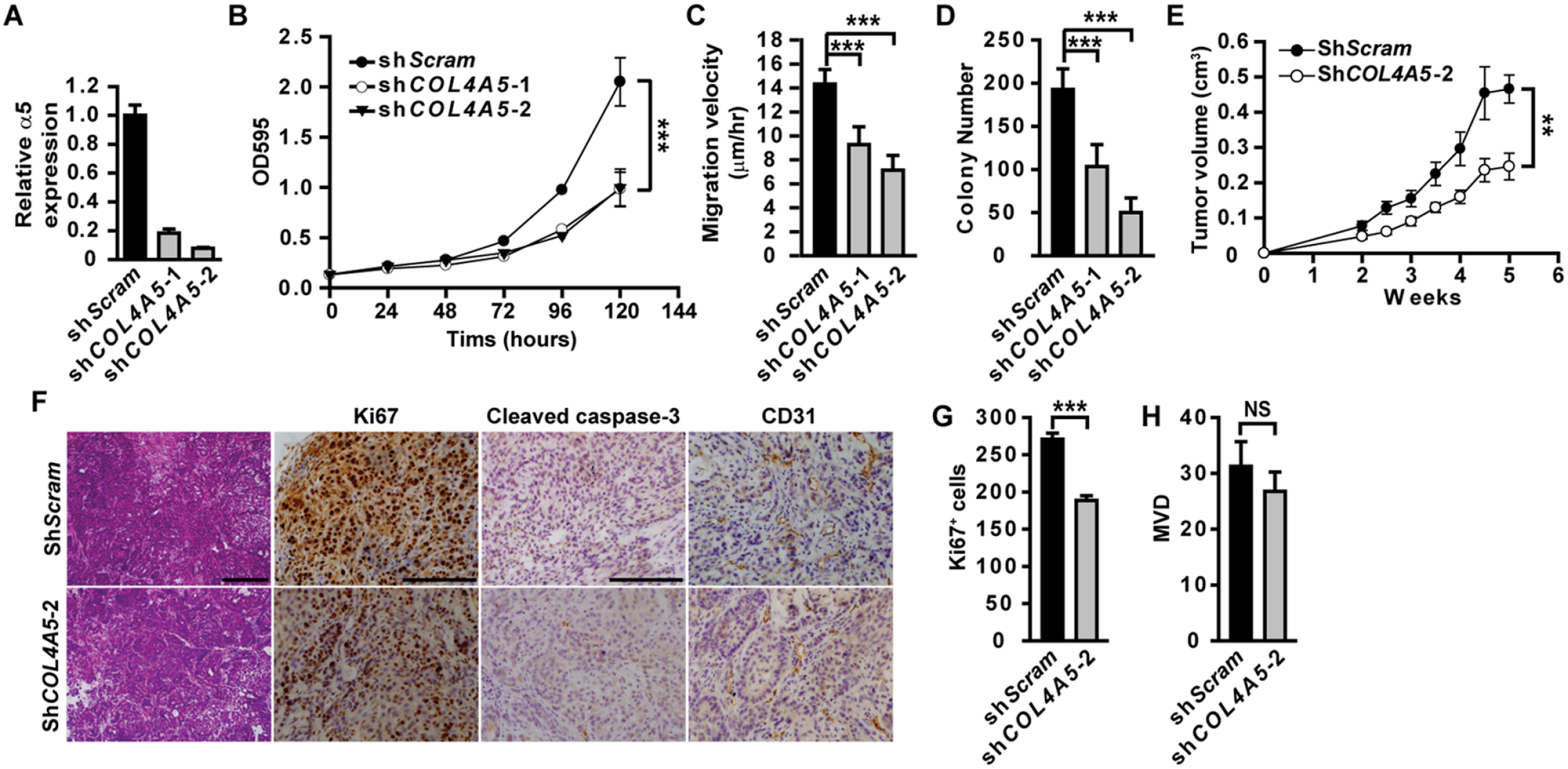
Epithelial α5(IV) supports tumor cell growth and tumorigenicity. (A) RT-PCR analysis of α5(IV) knockdown efficiency in A549 cells. (B-D) Knockdown of α5(IV) in A549 cells significantly impaired cell proliferation (B), migration (C) and anchorage-independent cell growth (D). Data are presented as mean ± SD. (E) Growth kinetics of xenograft tumors from A549 cells expressing scramble control (sh*Scram*) (n = 5) or *COL4A5* (sh*COL4A5*-2) (n = 6) shRNAs. (F) H&E, anti-Ki67, anti-cleaved caspase-3, or anti-CD31 immunohistochemical staining on xenograft tumor sections. No apoptotic signal was evident in both groups. (G and H) Quantitative proliferative indices (G) and microvascular density (MVD) (H) were measured. Data are presented as mean ± SEM. **P < 0.01, ***P < 0.001. NS: not significant. Scale bars: 200μm.

α5(IV) is expressed in lung bronchia and alveolar epithelial cells (S1 Fig). To study the functions of epithelial α5(IV) in lung cancer development, endogenous α5(IV) was knocked down in A549 lung adenocarcinoma cells (Fig 2A). α5(IV) knockdown significantly reduced A549 cell proliferation, migration and anchorage-independent cell growth (Fig 2B–2D), compared to cells expressing scramble control shRNA. This is not due to the off-target effect of α5(IV) shRNAs, as expression of mouse α5(IV) could rescue the phenotypes of α5(IV)-knockdown A549 cells (S2 Fig). α5(IV) knockdown in CRL-5810 lung cancer cells similarly resulted in impaired cell proliferation and anchorage-independent cell growth (S3 Fig). Therefore, the endogenous α5(IV)-constituted BMs are essential in supporting lung cancer cell proliferation. To determine whether in vitro phenotypes were reflected in vivo, tumorigenic ability of A549 cells was tested by injecting control or α5(IV)-knockdown cells subcutaneously into nude mice. α5(IV) knockdown resulted in slower growing A549 xenograft tumors (Fig 2E). Less proliferating cells were detected in α5(IV)-knockdown xenograft tumors (Fig 2F and 2G).

### α5(IV) is expressed in endothelial cells and regulates angiogenesis

**Fig 3:**
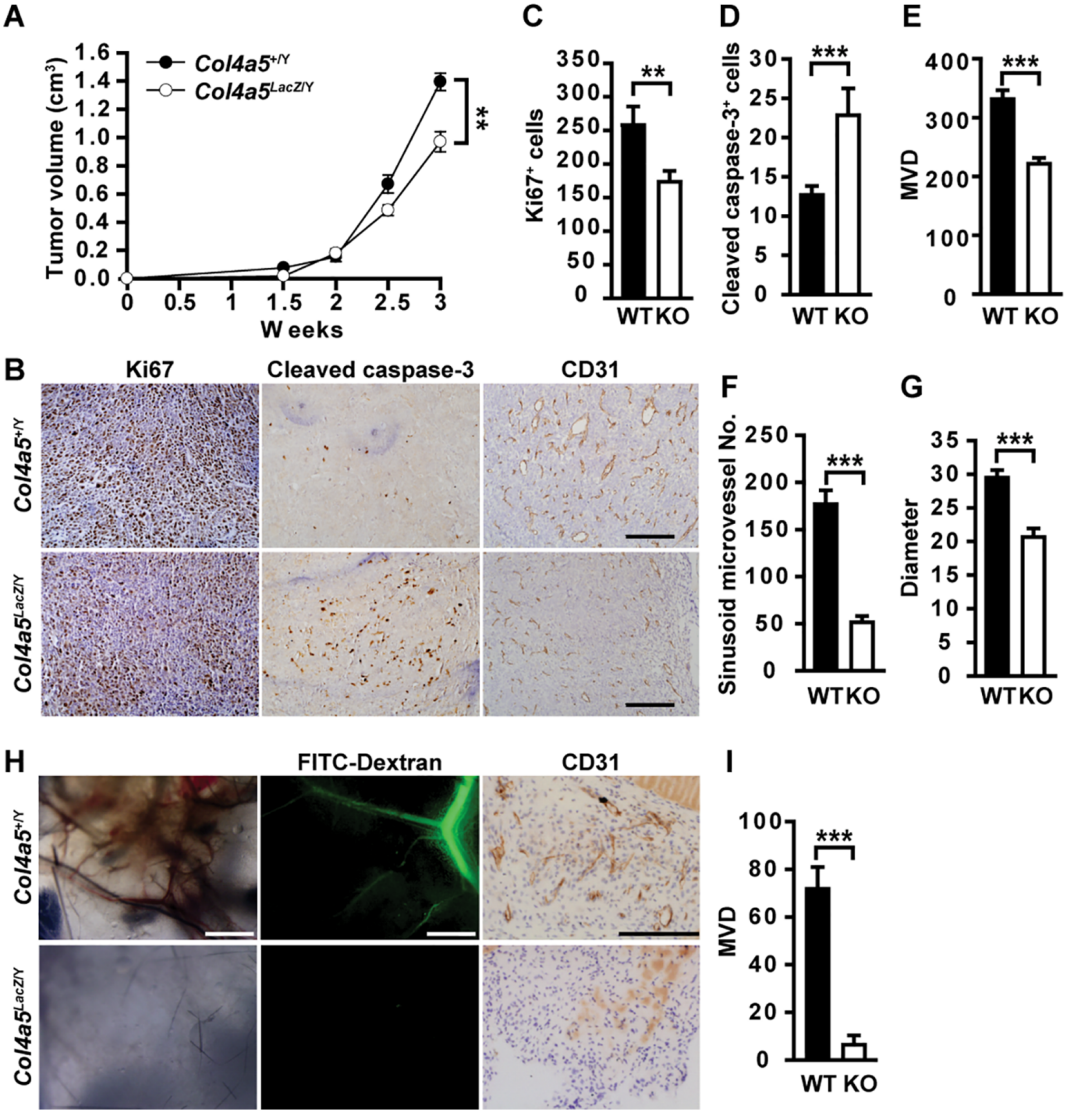
Stromal α5(IV) is required for tumor growth and tumor angiogenesis. (A) Growth kinetics of Lewis lung cancer cell (LLC) tumors transplanted in *Col4a5* KO mice (n = 6) or the WT littermates (n = 7). (B) Anti-Ki67, anti-cleaved caspase-3 or anti-CD31 immunohistochemical staining on LLC transplanted tumor sections implanted in WT or *Col4a5* KO mice. (C and D) Quantitative proliferative (C) and apoptotic (D) indices in LLC transplanted tumor sections on WT or KO mice (n = 5). (E-G) Quantitative microvascular density (MVD) (E), sinusoid microvessel number (VN) (F) and vascular diameter (G) in LLC tumor sections transplanted in WT or *Col4a5* KO mice (n = 5). (H) In vivo Matrigel plug assay in 8-week-old WT or *Col4a5* KO mice (n = 5). Dextran-FITC was injected through the tail vein to visualize the penetrating blood vessels (middle panels). Matrigel plugs were removed and fixed for CD31 staining (right panels). (I) Quantitative microvascular density (MVD) in Matrigel plugs implanted in WT or *Col4a5* KO mice (n = 5). Data are presented as mean ± SEM. **P < 0.01, ***P < 0.001. Scale bars: 200 μm.

**Fig 4:**
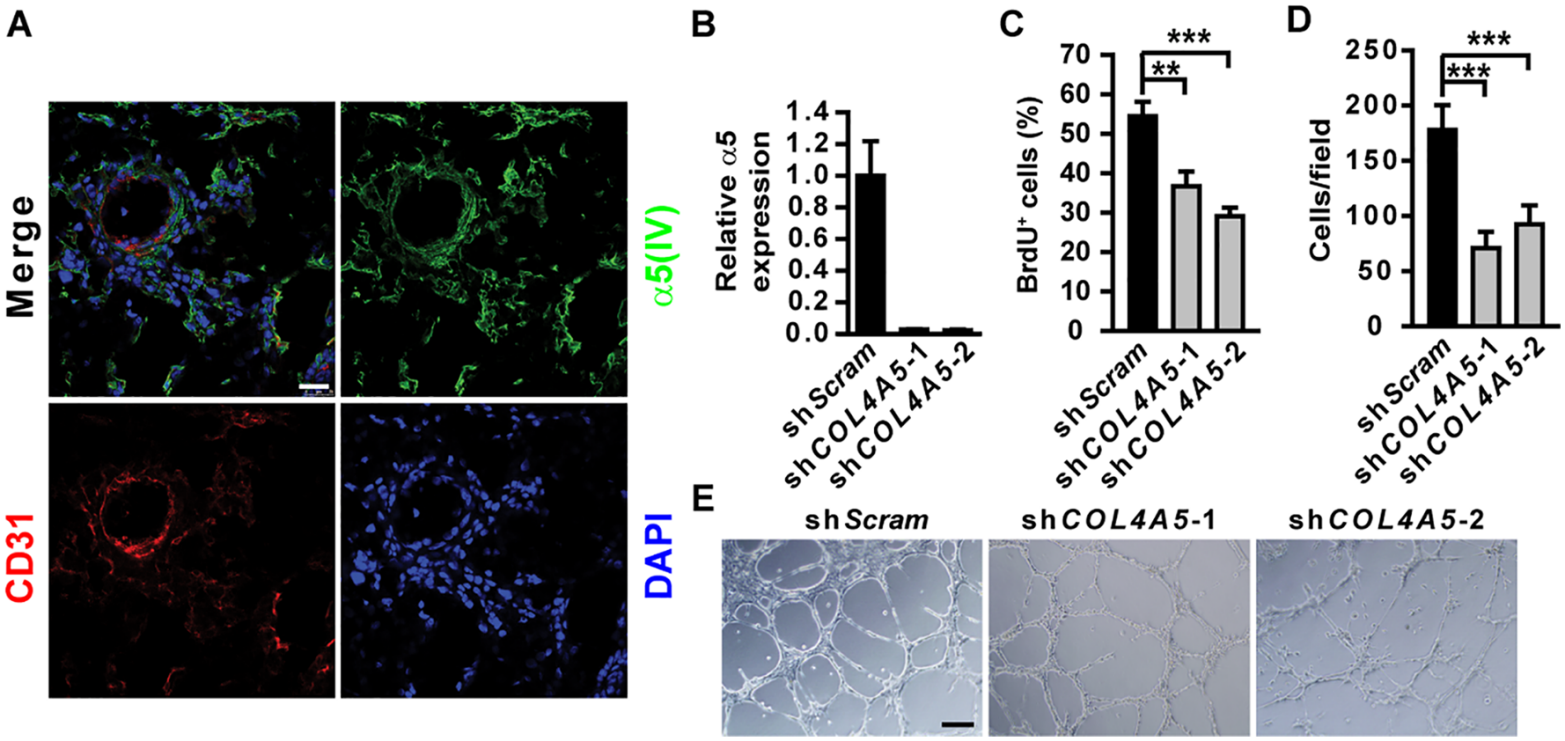
α5(IV) is expressed in endothelial cells and required for endothelial cell proliferation and tubulogenesis. (A) Immunofluorescent staining on lung sections shows partial colocalization of α5(IV) and CD31. Scale bar: 25μm. (B) RT-PCR analysis of α5(IV) knockdown efficiency in HMEC-1 cells. (C and D) Knockdown of α5(IV) impaired proliferation (C) and migration (D) of HMEC-1 cells, determined by BrdU incorporation and modified Boyden chamber assays, respectively. Data are presented as mean ± SD. ***P < 0.001. (E) In vitro tubulogenesis of HMEC-1 cells expressing control (sh*Scram*) or *COL4A5* (sh*COL4A5*) shRNAs. Scale bar: 200 μm.

Kras/α5 KO tumors were significantly less vascularized (Fig 1). However, knockdown of α5(IV) in A549 cells only mildly affected neo-angiogenesis in the xenograft tumors, which was not statistically significant (Fig 2F and 2H). This suggests that less angiogenesis observed in Kras/α5 KO tumors may be mainly due to ablation of stromal α5(IV). To examine the roles of stromal α5(IV) in tumor progression, murine Lewis lung cancer (LLC) cells were implanted in *Col4a5* WT or KO mice. Tumors grew significantly slower in KO than in WT mice (Fig 3A). Less proliferating cells were detected in the tumors from KO mice, than in that from WT mice (Fig 3B and 3D). Unlike the Kras-driven lung tumors, which were slowly growing and rare apoptosis was evident (Fig 1E), the LLC transplant tumors grew much faster. Apoptosis was evident in the LLC transplant tumors, due to rapid tumor growth (Fig 3C). More apoptotic cells were detected in the tumors from KO mice, than in that from WT mice (Fig 3C and 3D). These data collectively suggest stromal α5(IV) provides necessary survival and proliferation cues to support rapid LLC tumor growth. Tumors trigger profound angiogenesis to support vast nutrient and oxygen demand during rapid LLC transplant tumor growth in WT mice (Fig 3B). Fewer blood vessels formed in the LLC transplant tumors in the KO mice, compared to that in the WT mice (Fig 3B). The impaired tumor angiogenesis in the KO mice was not only reflected by decreased number of CD31-positive endothelial cells (Fig 3E), but also by dramatically decreased number of sinusoid microvessels (Fig 3F) and average vessel diameter (Fig 3G). To further test if stromal α5(IV) plays a role in regulating angiogenesis, VEGF containing Matrigel plugs were implanted subcutaneously in *Col4a5* WT or KO mice. Abundant blood vessels, visualized by FITC-dextran, formed in the Matrigel plugs implanted in the WT mice, but not in the KO mice (Fig 3H). CD31 staining on Matrigel plug sections further revealed ~12-fold reduction of capillary numbers in the plugs in KO mice (Fig 3H and 3I). α5(IV) partially colocalized with endothelial cell marker CD31 in the lung (Fig 4A). Knockdown of α5(IV) in human microvascular endothelial cell-1 (HMEC-1) cells (Fig 4B) significantly reduced endothelial cell proliferation (Fig 4C) and migration (Fig 4D). Knockdown of α5(IV) in HMEC-1 cells also significantly impaired the tubule formation capability of endothelial cells (Fig 4E). Thus, endothelial α5(IV) may be responsible for efficient tumor angiogenesis.

### α1(IV) can not functionally compensate for α5(IV)

**Fig 5:**
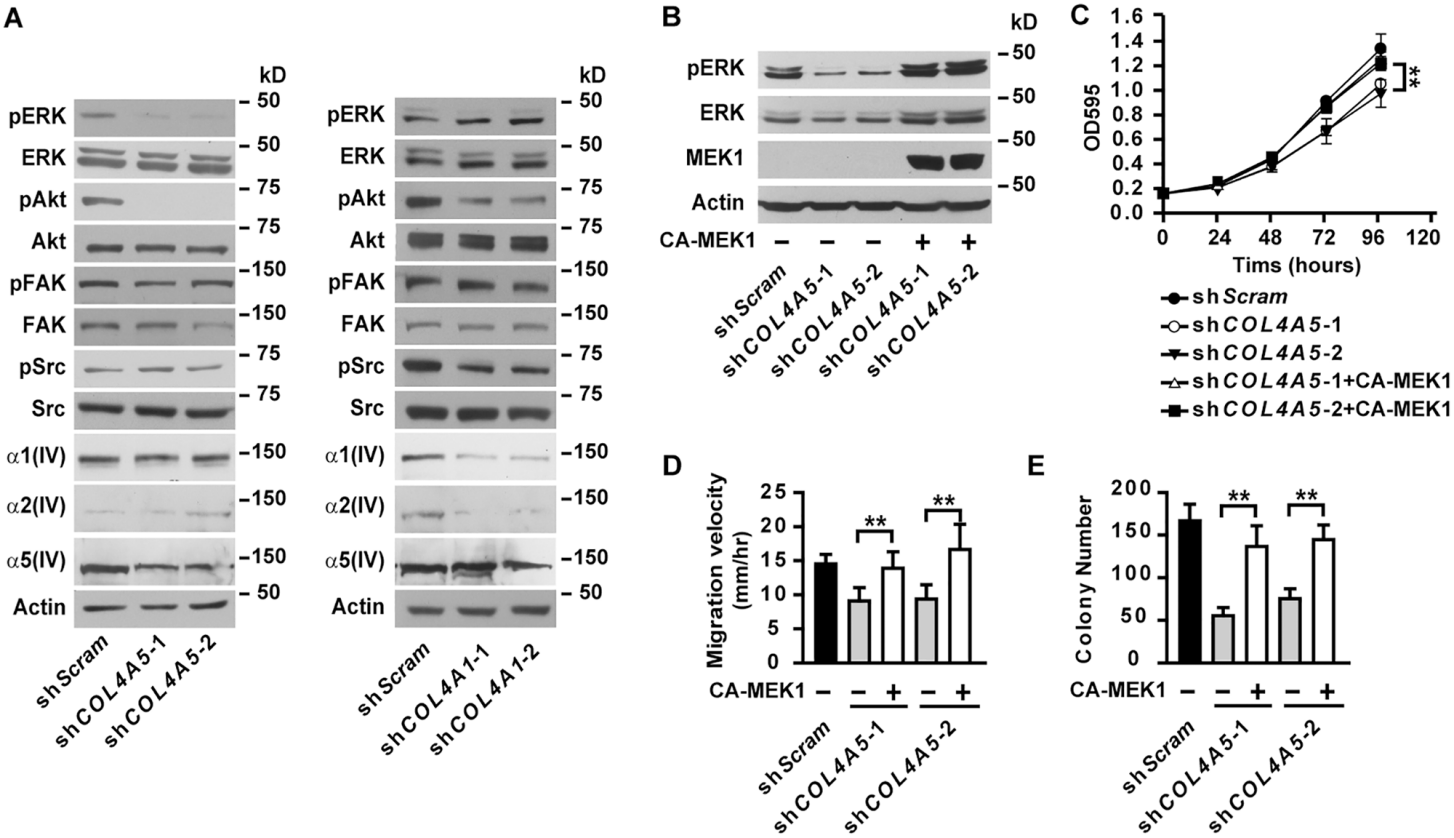
α5(IV), but not α1(IV), deficiency results in impaired activation of ERK. (A) Western blot analyses of phosphorylation levels of ERK, Akt, FAK and Src and α1(IV), α2(IV) and α5(IV) expression in α5(IV)- or α1(IV)-knockdown A549 cells. (B) Western blot analyses of phosphorylation levels of ERK in α5(IV)-knockdown A549 cells expressing constitutively active MEK1. (C-E) Expression of constitutively active MEK1 in α5(IV)-knockdown A549 cells rescued the defects in cell proliferation (C), migration (D), and anchorage-independent cell growth (E). Data are presented as mean ± SD. **P < 0.01.

Major Col IV is known to provide survival and growth cues to cancer cells. α5(IV) may regulate tumor progression through modulating major Col IV expression and basement membrane assembly. Electron microscopy on the lungs from 6-month old KO mice did not reveal overt defect in the basement membranes underneath lung alveolar epithelial cells (S4A Fig). Relatively more abundant α1(IV) expression was detected in KO lungs, compared to WT tissues (S4B Fig). Ablation of α5(IV) had no significant effect on α1(IV) expression in Kras-driven lung tumors (S4C Fig). Knockdown of α5(IV) in A549 (Fig 5A) and HMEC-1 (S7A Fig) cells did not significantly affect major Col IV α1(IV) or α2(IV) chain expression. Despite knockdown of α1(IV) impaired cellular functions of A549 (S5 Fig) and HMEC-1 (S6 Fig) cells, expression of α5(IV) was not affected (Fig 5A and S7A Fig). All these data collectively suggest that altered behavior of α5(IV)-deficient cells and impaired tumor progression in α5(IV)-deficient mice are not the results of concomitant loss of major Col IV expression or disruption of basement membrane structure. The presence of abundant α1(IV) also suggests that major Col IV can not functionally compensate for the loss of α5(IV) in supporting tumor growth.

### Loss of α5(IV) impaired ERK activation

FAK is one of the major effecters transducing signals from Col IV [14]. FAK further phosphorylates and activates downstream signaling molecules, including Src [14]. Knockdown of α5(IV), however, did not affect phosphorylation levels of FAK and Src in A549 and CRL-5810 lung cancer cells (Fig 5A and S3A Fig). Instead, significantly lower phosphorylation levels of ERK and Akt, kinases essential in supporting cell survival, proliferation and transformation [15,16], were detected in α5(IV)-knockdown A549 and CRL-5810 cells (Fig 5A and S3A Fig). Ectopic expression of mouse α5(IV) in α5(IV)-knockdown A549 cells restored phosphorylation of ERK and Akt (S2E Fig). Interestingly, knockdown of α1(IV) resulted in impaired phosphorylation of Akt and Src, but not ERK or FAK in A549 cells (Fig 5A), reinforcing the notion that major and minor Col IV may regulate cancer cell behavior through overlapping, but not identical intracellular signaling pathways. Similar to that in lung cancer cells, knockdown of α5(IV), but not α1(IV), significantly decreased ERK phosphorylation in HMEC-1 cells (S7A Fig). To study if impaired ERK activation is responsible for the defects in cell proliferation and migration resulted from α5(IV) deficiency, constitutively active MEK1 was expressed in α5(IV)-knockdown A549 and HMEC-1 cells. Expression of constitutively active MEK1 successfully restored ERK phosphorylation in A549 and HMEC-1 cells (Fig 5B and S7B Fig). Expression of constitutively active MEK1 in A549 cells rescued the defects of cell proliferation (Fig 5C), migration (Fig 5D) and anchorage-independent cell growth (Fig 5E). Constitutively active MEK1 also restored the capability of cell proliferation (S7C Fig), migration (S7D Fig) and tubule formation (S7E Fig) of α5(IV)-knockdown HMEC-1 cells.

### DDR1 transduces signal from α5(IV)

**Fig 6:**
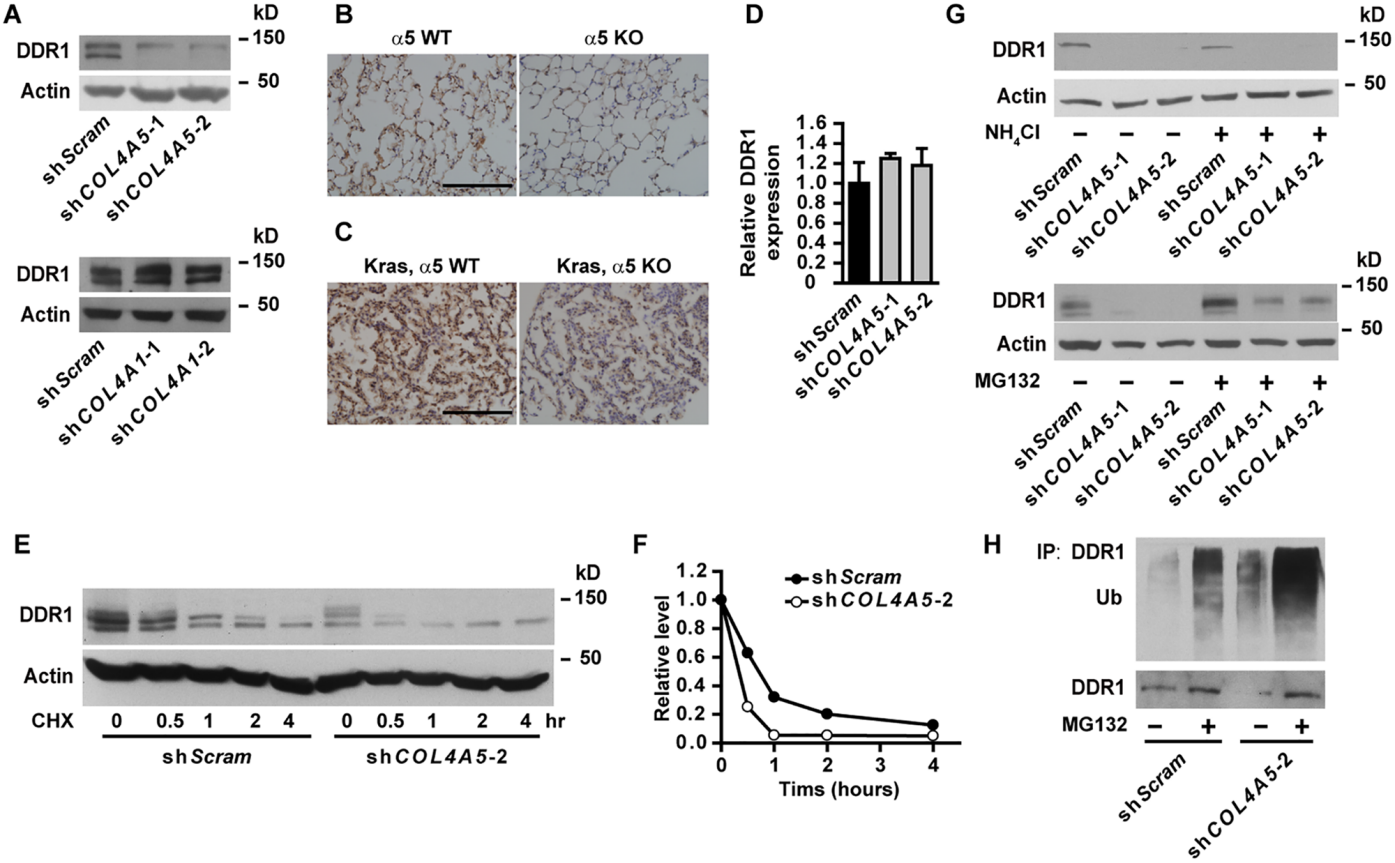
DDR1 is downregulated in α5(IV)-knockdown cells. (A) Western blot analyses of DDR1 expression in α5(IV)- or α1(IV)-knockdown A549 cells. (B and C) DDR1 staining on lung sections from *Col4a5* ^+/Y^ and *Col4a5* ^*LacZ*/Y^ mice (B) or lung tumor sections from Kras/α5 WT and Kras/α5 KO mice (C). Scale bars: 200 μm. (D) Quantitative RT-PCR analysis of DDR1 expression in A549 cells expressing control (sh*Scram*) or *COL4A5* (sh*COL4A5*) shRNAs. (E and F) A549 cells expressing control (sh*Scram*) or *COL4A5* (sh*COL4A5*) shRNAs were treated with or without 100 μg/mL cycloheximide (CHX) for 0.5, 1, 2 or 4 hours. (E) DDR1 protein levels were analyzed by western blot. (F) Relative protein levels of DDR1 after cycloheximide treatment in (E). (G) Western blot analysis of DDR1 expression in A549 cells expressing control (sh*Scram*) or *COL4A5* (sh*COL4A5*) shRNAs treated with or without 10 μM proteasome inhibitor MG132 for 4 hours or 50 mM lysosome inhibitor NH_4_Cl for 6 hours. (H) A549 cells expressing control (sh*Scram*) or *COL4A5* (sh*COL4A5*) shRNAs were treated with 10 μM proteasome inhibitor MG132 for 4 hours. DDR1 was immunoprecipitated and ubiquitination levels were detected.

Col IV transduces signals through cell surface integrin and non-integrin receptors [5]. Knockdown of α5(IV) had no effect on cell surface integrin expression (S8 Fig). Knockdown of α5(IV) significantly decreased the expression of non-integrin collagen receptor DDR1 in lung cancer cells (Fig 6A and S3A Fig), which can be restored by ectopic mouse α5(IV) expression (S2E Fig). However, DDR1 expression was not altered in α1(IV)-knockdown lung cancer cells (Fig 6A). Similar to that in lung cancer cells, DDR1 expression was decreased in α5(IV)-, but not α1(IV)-, knockdown HMEC-1 cells (S9A Fig). In addition, significantly less DDR1 expression was detected in KO lungs, compared to WT tissues (Fig 6B). Ablation of α5(IV) also significantly decreased DDR1 expression in Kras-driven lung tumors (Fig 6C). Interestingly, α5(IV) knockdown in A549 cells did not affect DDR1 mRNA levels (Fig 6D), suggesting α5(IV) ablation may regulate DDR1 expression via mechanisms other than transcriptional regulation. A much faster decline of DDR1 protein was observed in α5(IV)-knockdown A549 cells subjected to cycloheximide treatment (Fig 6E and 6F), suggesting that α5(IV) regulates DDR1 expression at least partially by stabilizing DDR1 proteins. Lysosome inhibitor NH_4_Cl had minimal effect on DDR1 protein levels (Fig 6G). Proteasome inhibitor MG132 treatment restored DDR1 protein levels in α5(IV)-knockdown A549 cells (Fig 6G). α5(IV) knockdown significantly increased DDR1 ubiquitination in A549 cells (Fig 6H). Therefore, α5(IV) ablation downregulates DDR1 expression by accelerating DDR1 ubiquitination and proteasome-dependent degradation.

**Fig 7:**
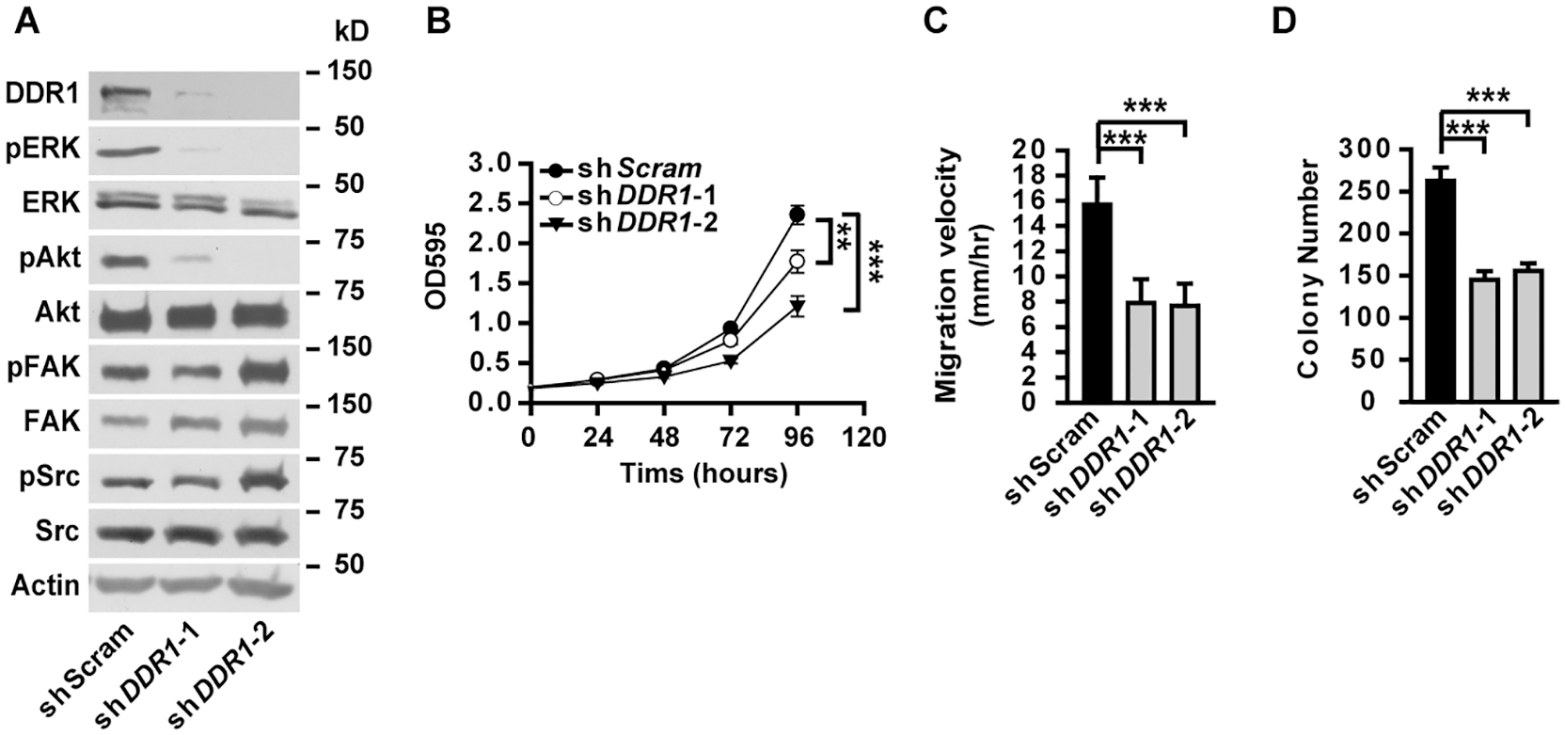
DDR1 is required for ERK activation, cell proliferation and migration in lung cancer cells. (A) Western blot analyses of phosphorylation levels of ERK, Akt, FAK and Src in A549 cells with DDR1 knockdown. (B-D) Knockdown of DDR1 in A549 cells significantly impaired cell proliferation (B), migration (C), and anchorage-independent cell growth (D). Data are presented as mean ± SD. **P < 0.01, ***P < 0.001.

**Fig 8:**
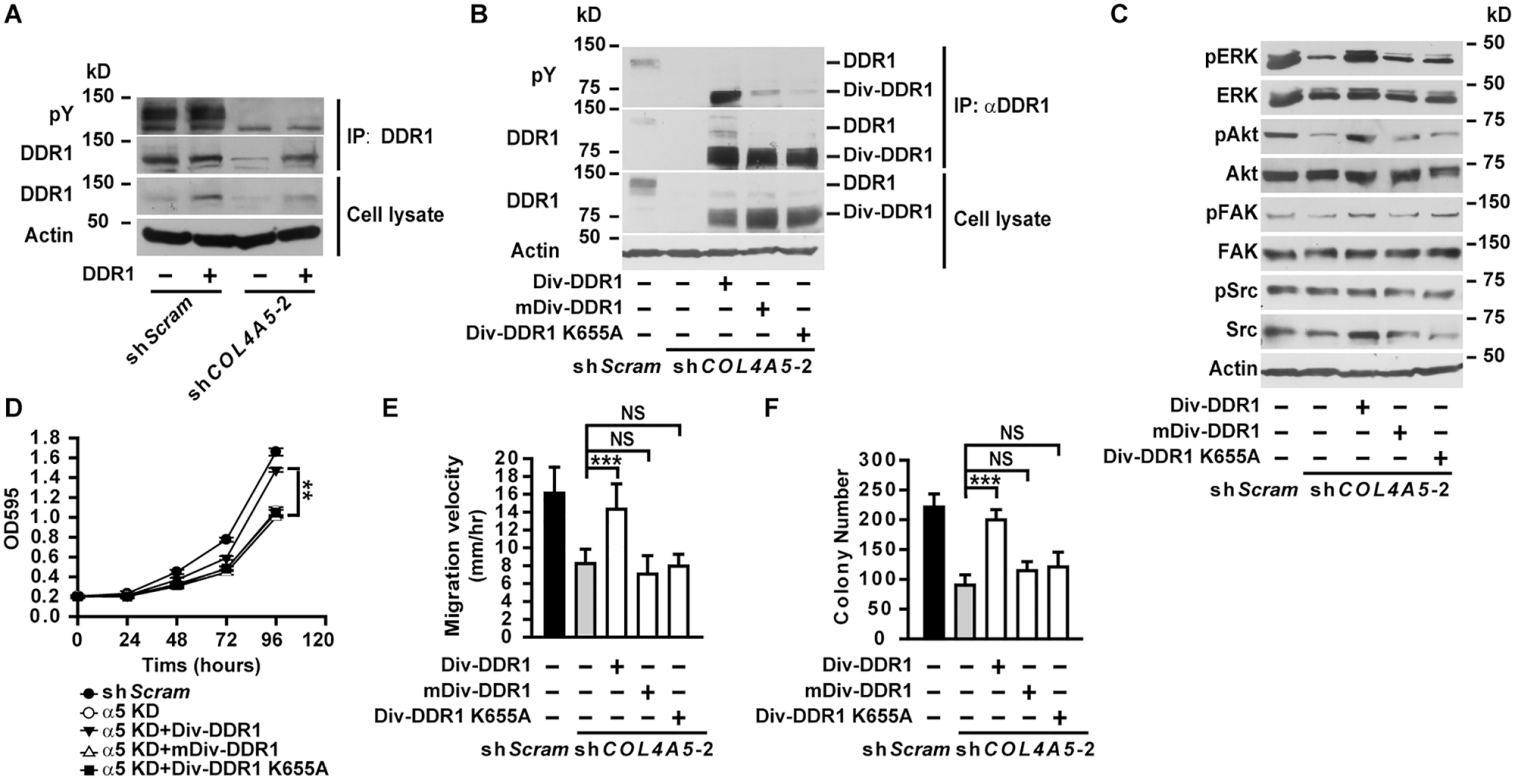
Constitutively active DDR1 rescued the defects of α5(IV)-deficient lung cancer cells. (A) Full-length wild-type DDR1 was expressed in control or α5(IV)-knockdown A549 cells. DDR1 was immunoprecipitated and tyrosine phosphorylation levels were detected. (B) Div-DDR1 chimeric proteins were expressed in α5(IV)-knockdown A549 cells. DDR1 expression and tyrosine phosphorylation levels were detected. Div-DDR1 with mutations in the Div coil-coiled domain (mDiv-DDR1) or kinase domain (Div-DDR1 K655A) failed to activate and autophosphorylate. (C) Western blot analyses of phosphorylation levels of ERK, Akt, FAK and Src in α5(IV)-knockdown A549 cells expressing Div-DDR1 chimeric proteins. (D-F) Expression of Div-DDR1, but not mDiv-DDR1 or Div-DDR1 K655A in α5(IV)-knockdown A549 cells rescued the defects in cell proliferation (D), migration (E), and anchorage-independent cell growth (F). Data are presented as mean ± SD. ***P < 0.001. NS: not significant.

Knockdown of DDR1 in A549 cells resulted in decreased phosphorylation of ERK and Akt (Fig 7A), unaffected phosphorylation of FAK and Src (Fig 7A), as well as impaired cell proliferation (Fig 7B), migration (Fig 7C) and anchorage-independent cell growth (Fig 7D), resembling the phenotypes observed in α5(IV)-knockdown A549 cells. Knockdown of DDR1 in HMEC-1 cells similarly resulted in decreased phosphorylation of ERK and Akt (S9B Fig), impaired endothelial cell proliferation (S9C Fig), migration (S9D Fig) and tubule formation (S9E Fig). The similar phenotypes observed in the α5(IV)- and DDR1-knockdown cells indicate that DDR1 may be the receptor transducing signals from α5(IV). DDR1 is a receptor tyrosine kinase that its phosphorylation is indicative of receptor activation and important in transducing downstream signals. Significantly less phosphorylation of DDR1 was detected in α5(IV)-knockdown A549 cells, compared to that in cells expressing scramble shRNA (Fig 8A). DDR1 expression was reduced in α5(IV)-knockdown cells and less amount of DDR1 was immunoprecipitated (Fig 8A). To more accurately examine DDR1 phosphorylation levels in α5(IV)-knockdown cells, DDR1 was expressed back to endogenous levels. Less DDR1 phosphorylation was detected in α5(IV)-knockdown A549 cells expressing exogenous DDR1 than the control cells, despite similar amount of DDR1 was immunoprecipitated (Fig 8A). Overexpression of DDR1 was not able to restore phosphorylation levels of ERK and Akt in α5(IV)-knockdown A549 cells (S10 Fig). These data collectively suggest that α5(IV) not only affects DDR1 stability and expression, but also is required for DDR1 activation.

To further study if DDR1 is functionally downstream of α5(IV), a chimeric Div-DDR1 is expressed in α5(IV)-knockdown A549 cells. The chimeric Div-DDR1 is constructed by replacing the extracellular ligand binding discoidin domain of DDR1 with Div, a coil-coiled domain from *Bacillus subtilis* DivIVA that forms constitutive dimer/oligomer [17,18]. Replacement of DDR1 ligand binding domain with Div provokes spontaneous DDR1 autophosphorylation and activation (Fig 8B) [18,19]. Expression of such a constitutively active Div-DDR1 in α5(IV)-knockdown A549 cells restored ERK and Akt phosphorylation (Fig 8C), cell proliferation (Fig 8D), migration (Fig 8E) and anchorage-independent cell growth (Fig 8F). Oligomerization capability and kinase activity of DDR1 are necessary for DDR1 function. Div-DDR1 with mutations in the Div coil-coiled domain (mDiv-DDR1) that disrupts Div self-assembly ability [17] or in the DDR1 kinase domain (Div-DDR1 K655A) that impairs DDR1 tyrosine kinase activity [20] failed to activate DDR1 (Fig 8B). Expression of such DDR1 mutants also failed to restore ERK and Akt phosphorylation (Fig 8C), cell proliferation (Fig 8D), migration (Fig 8E) and anchorage-independent cell growth (Fig 8F) in α5(IV)-knockdown A549 cells. To study if the DDR1 signaling pathway is involved in transducing signal from α5(IV) in endothelial cells, constitutively active DDR1 was expressed in α5(IV)-ablated HMEC-1 cells. Expression of constitutively active Div-DDR1, but not mDiv-DDR1 or Div-DDR1 K655A, in α5(IV)-knockdown HMEC-1 cells restored ERK and Akt phosphorylation (S11A Fig), and rescued the defects of cell proliferation (S11B Fig), migration (S11C Fig) and tubule formation (S11D Fig).

## Discussion

Col IV, the major BM component, is essential in maintenance of tissue integrity and proper function. In addition to broadly expressed and extensively studied major Col IV α1α1α2, minor Col IV α3α4α5 and α5α5α6 are less abundantly expressed with restricted tissue distribution [4]. Physiological and pathological functions of minor Col IV, however, are less well understood. In this report, we present evidences that minor Col IV α5(IV) is essential in supporting lung cancer development via cancer cell autonomous and non-autonomous mechanisms. Minor but not major Col IV signals through non-integrin receptor DDR1.

Delayed tumor progression in α5(IV)-deficient mice suggests proper signal from α5(IV) is important in supporting cancer cell survival and proliferation. Col IV transduces signals through cell surface receptors. Cell surface integrin expression is unaffected in α5(IV)-knockdown cells. However, expression of DDR1, the non-integrin collagen receptor functioning independent of integrins [20–22], is decreased in α5(IV)-knockdown cells. DDR1 is highly phosphorylated in non-small cell lung cancer (NSCLC) [23], and DDR1 overexpression is associated with poor prognosis in NSCLC [24]. Inhibition of DDR1 reduces cell survival, homing and colonization in lung cancer metastasis [25]. Consistently, DDR1 expression is elevated in lung tumors with Kras activation, compared to normal lung tissues (compare Fig 6B and 6C). Ablation of α5(IV) results in decreased DDR1 expression in both normal lung tissues and Kras lung tumors. DDR1-knockdown cells phenocopied α5(IV)-knockdown cells. More importantly, expression of constitutively active DDR1 in α5(IV)-knockdown cells can rescue the proliferation and migration defects, suggesting DDR1 is functionally downstream of α5(IV). α5(IV) knockdown impaired DDR1 phosphorylation. Overexpression of exogenous wild-type DDR1 can not restore ERK phosphorylation in α5(IV)-knockdown cells. These data indicate that the function of DDR1 requires the presence of α5(IV) and DDR1 may directly mediate the functions of α5(IV).

Despite α5(IV) knockdown does not affect integrin cell surface expression, the possibility exists that integrins are functional receptors for α5(IV). Col IV was reported to bind integrins through sites within the triple-helical cyanogen bromide-derived fragments and noncollagenous domains [5]. Such studies were largely based on purified Col IV or Col IV fragments. It should be noted that proper collagen network assembly and geometry are critical in the biological functions of Col IV. Ablation of endogenous Col IV using gene knockout or silencing will provide more physiologically relevant insights into receptor binding, signaling and biological functions of Col IV. It remains to be elucidated whether integrins have selectivity and specificity towards major and minor Col IV under different physiological and pathological circumstances. DDR1 and integrins may have cooperative or opposing functions in response to collagens [26,27]. The crosstalk between DDR1 and integrins upon α5(IV) binding may provide the cells more robustness.

Ablation of α5(IV) does not affect major Col IV expression, or disrupt basement membrane assembly. The inability of abundant major α1α1α2(IV) to support efficient tumor growth and progression in α5(IV)-deficient mice indicates that major Col IV can not functionally compensate for the deficiency of minor Col IV. This is supported by the fact that mutations of Col IV α chains cause distinct heritable diseases. Mutations in *COL4A1* cause encephaloclastic porencephaly, characterized by degenerative cavities and cerebral lesions in the brain [28]. Deletion of *Col4a1/Col4a2* locus in mice results in growth retardation and embryonic lethality [29]. However, mutations in *COL4A5* (Alport’s syndrome) or auto-antibody recognizing α3(IV) (Goodpasture’s syndrome) result in progressive renal failure [4,5]. Mice deficient of α3(IV) [30,31] or α5(IV) [32] are viable, but develop renal phenotypes reminiscent of that in Alport’s syndrome. Knockdown of major Col IV α1(IV) does not affect DDR1 expression. The overlapping, but not identical spectrum of altered signaling events in α5(IV)- and α1(IV)-knockdown cells suggests that major and minor Col IV may exert their biological functions via different cell surface receptors and intracellular signaling pathways.

Major and minor Col IV share same domain structure and high sequence similarity. It is yet unclear how highly similar major and minor Col IV recognize different cell surface receptor and activate different intracellular signaling pathways. α3α4α5(IV) is highly cross-linked due to its larger degree intra- and inter-chain disulfide bonds, relative to α1α1α2(IV) [33]. As a result, α3α4α5(IV) has different biochemical properties from α1α1α2(IV) that α3α4α5(IV) is more resistant to proteolytic degradation [33]. Different biomechanical force from major and minor Col IV may be responsible for the receptor specificity. It should be noted that Col IV protomers further form α1α1α2(IV)-α1α1α2(IV), α3α4α5(IV)-α3α4α5(IV) and α1α1α2(IV)-α5α5α6(IV) networks [4,5]. These networks may differentially recognize cell surface receptors and activate intracellular signaling pathways, thus provide signal specificity and redundancy.

α5(IV) regulates cancer progression via cancer cell autonomous and non-autonomous mechanisms. The DDR1-ERK signaling cascade is required for the functions of both cancer cells and endothelial cells. Stromal components, including blood vessels, constitute proper microenvironment to support tumor progression. It is reported that stable microvasculature sustains cancer cells at dormancy, whereas sprouting neovasculature rescues cancer cells from cell cycle arrest and promotes cancer cell proliferation [34]. Col IV assembly is critical for vascular BM integrity and structural organization. Small-molecule inhibitors that interfere Col IV biosynthesis were shown to prevent angiogenesis and tumor growth [35]. α5(IV) is expressed in the endothelium. Deficiency of α5(IV) delayed in vitro and in vivo angiogenesis. It warrants further study if the cancer cells in α5(IV) KO mice remain dormant due to impaired neo-angiogenesis.

In summary, we provides evidences in this study that α5(IV) deficiency significantly delays tumor progression. α5(IV) signals through non-integrin collagen receptor DDR1 in lung cancer cells and endothelial cells. α5(IV) promotes tumor growth via both cancer cell autonomous and non-autonomous mechanisms. Abundant major Col IV is not able to compensate for α5(IV) deficiency.

## Materials and Methods

### Ethics statement

All mice were housed in specific pathogen-free environment at the Shanghai Institute of Biochemistry and Cell Biology and treated in strict accordance with protocols approved by the Institutional Animal Care and Use Committee of Shanghai Institute of Biochemistry and Cell Biology (Approval number: SIBCB-NAF-15-003-S325-006).

### Reagents

The antibodies used are ERK, ERK pT202/pY204, Akt, Akt pS473, Src, Src pY416, cleaved caspase-3 (Cell Signaling), FAK (BD Transduction Laboratories), FAK pY397 (Millipore), Ki-67 (Novocastra Laboratories), α1(IV) (Abgent), α2(IV) and CD31 (Abcam), α5(IV) (rabbit ployclonal antibody from Proteintech (western blot) and rat monoclonal antibody clone b14 (immunostaining) provided by Dr. Yoshikazu Sado, Shigei Medical Research Institute [36]), DDR1, phosho-tyrosine (pY99), ubiquitin (Santa Cruz), MEK1 (Abmart), Actin (Sigma-Aldrich), biotinylated goat anti-rabbit secondary antibody (Zymed), Alexa Fluor 555/488 conjugated anti-mouse, rat or rabbit IgG secondary antibodies (Invitrogen). Expression level of integrins on A549 cell surface was determined by immunofluorescence flow cytometry with anti-β1 (Thermo Scientific Pierce), α1, α2 and α11 (Santa Cruz) integrin antibodies as described [37]. Cycloheximide, MG132 and NH_4_Cl were purchased from Sigma-Aldrich.

### Plasmids and generation of stable cell lines

The shRNAs were cloned into pLKO.1-puro lenti-viral vector (Addgene). Viral packaging and infection of cells was performed as previously described [38]. After viral infection, cells were selected with puromycin to generate stable cell lines. At least two batches of stable cell lines were generated for each experiment. Experiments were performed in triplicates and repeated at least twice using each batch of cells. The target sequences are: 5’-CAACAAGATGAAGAGCACCAAC-3’ (sh*Scram*), 5’-GGGTGATGATGGAATTCCA-3’ (sh*COL4A5*-1), 5’-GCAGATCAGTGAACAGAAAAG-3’ (sh*COL4A5*-2), 5’-TCCAGGATGCAATGGCACAAA -3’ (sh*COL4A1*-1), 5’-TCCAGGTTCCAAGGGAGAAAT -3’ (sh*COL4A1*-2), 5’-GGTTACTCTTCAGCGAAAT -3’ (sh*DDR1*-1), and 5’-AGATGGAGTTTGAGTTTGACC -3’ (sh*DDR1*-2).

To generate cell lines expressing mouse α5(IV), DDR1 or Div-DDR1, coding sequences were cloned into pCDH-Neo lenti-viral vector (Addgene). α5(IV)-knockdown cells were infected with lenti-virus harboring mouse α5(IV), DDR1 or Div-DDR1 sequences and selected with G418. Mouse *Col4a5* sequences were amplified from B16-F10 cDNA using primers 5’-gatcTCTAGAatgcaagtgcgtggagtgtgcc-3’ (forward) and 5’-gatcGCGGCCGCttatgtcctcttcatgcatact-3’ (reverse). Amplicon was inserted into pCDH-Neo vector. HA-tagged human DDR1 was cloned from MCF-7 cDNA using primers 5’-GATCGAATTCATGGGACCAGAGGCCCTGT-3’ (forward) and 5’-GATCGCGGCCGCTCAAGCGTAATCTGGAACATCGTATGGGTACACCGTGTTGAGTGCATCCT-3’ (reverse). Amplicon was inserted into pCDH-Neo. K655A substitution was introduced by two step PCR amplification that was restricted with XhoI and NotI and exchanged for the corresponding wild-type fragment in the DDR1 expression construct. The primers used were 5’-CCCGTCCCCCTCGAGGCCC-3’ (fragment 1, forward), 5’-CCGTAAGATCGCGACAGCTACCAGCAAAGG-3’ (fragment 1, reverse), 5’-GTAGCTGTCGCGATCTTACGGCCAGATGCC-3’ (fragment 2, forward), and 5’-GATCGCGGCCGCTCAAGCGTAATCTGGAACATCGTATGGGTACACCGTGTTGAGTGCATCCT-3’ (fragment 2, reverse). To generate the Div-DDR1 chimeric proteins, the coding sequences of DDR1 discoidin domain (aa 29–367) was replaced by a 51bp oligonecleotide sequences compromising BstBI and BamHI restriction sites by two step PCR. The primers used were 5’-GATCgaattcATGGGACCAGAGGCCCTGT-3’ (fragment 1, forward), 5’-GGATCCGTGATAGTTTTTGCTAAGCAACTCTTCAACTTTATCTTCCAACTGTTTCATTTCGAACTTGGCAGGATCAAAATGTC-3’ (fragment 1, reverse), 5’- TTCGAAATGAAACAGTTGGAAGATAAAGTTGAAGAGTTGCTTAGCAAAAACTATCACGGATCCGTGGTGAACAATTCCTCTCCG-3’ (fragment 2, forward), and 5’-GATCgcggccgcTCAAGCGTAATCTGGAACATCGTATGGGTACACCGTGTTGAGTGCATCCT-3’ (fragment 2, reverse). The Div coil-coiled domain [18] (wild-type: MKQLEDKVEELLSKNYHLENEVARLKKLVGERGSSGSGR; mutant: MKQLEDKVEELLSKNYHVENEVARVKKLVGERGSSGSGR), amplified using primers 5’-GATCTTCGAAATGAAACAGTTGGAAGATAAAG-3’ (forward) and 5’-GATCGGATCCGCGGCCGCTTCCAGAGCTTCC-3’ (reverse), was placed between BstBI and BamHI sites. Human CA-MEK1 was prepared by substituting Ser 218 and Ser 222 in MEK1 with glutamic acids and removing residues 31 to 52 as described [39].

### RT-PCR

Total RNA was prepared and retrotranscribed as described [40]. The RT-PCR primers used are: human/mouse *COL4A5*: 5’-TGCCTTCGTCGCTTTAGT-3’ (forward) and 5’-TTGACCTGAGCCTTCTGC-3’ (reverse); Mouse *Col4a5*: 5’-GGATTGGCTATTCCTTCAT-3’ (forward) and 5’-GCATACTTGACATCGGCTA-3’ (reverse); Human/mouse *ACTIN*: 5’-cctagaagcatttgcggtgg-3’ (forward) and 5’-gagctacgagctgcctgacg-3’ (reverse).

### Cell proliferation, migration and anchorage-independent cell growth assays

A549 and CRL-5810 cells (ATCC) were maintained in RPMI 1640 (Hyclone) supplemented with 5% FBS (Biochrom). 293T cells and Lewis lung cancer (LLC) cells (ATCC) were cultured in DMEM (GIBCO) with 10% FBS. Human microvascular endothelial cell-1 (HMEC-1) (generously provided by Dr. Zhengjun Chen) was cultured in MCDB131 (GIBCO) with 10% FBS, 10ng/mL EGF and 1μg/mL hydrocortisone. To study the functions of endogenous Col IV, the lung cancer cells and endothelial cells were plated directly on tissue culture plates without exogenous substance coating. MTT, bromodeoxyuridine (BrdU) incorporation, migration and anchorage-independent cell growth assays were performed as described [40,41].

### In vitro and in vivo angiogenesis assay

In vitro angiogenesis assay was performed as described [42] by seeding HMEC-1 cells in the rat tail type I collagen sandwich gel in the presence of VEGF. Cells were photographed after 24 hours. In vivo Matrigel plug assay was performed as described [43] by subcutaneously injecting growth factor reduced Matrigel containing 50 ng recombinant human vascular endothelial growth factor into 8-week-old WT or *Col4a5* ^*LacZ*/Y^ mice in C57/Bl background. On day 14, Dextran-FITC was injected through the tail vein 30 min before the mice were sacrificed. Matrigel plugs were fixed and sectioned for CD31 staining. Histological vascular parameters, including microvascular density (MVD), sinusoid microvessel number, and vascular diameter, were measured [44].

### Immunoprecipitation and immuno blot

Total cell lysates were harvested in hot SDS sample buffer. For immunoprecipitation, cells were lysed in RIPA buffer. DDR1 was immunoprecipitated with anti-DDR1 (Santa Cruz) antibody. Immunoprecipitated proteins were eluted with SDS sample buffer. Proteins were separated by SDS-PAGE. After electrophoresis, the proteins were transferred to nitrocellulose membrane. The membrane was incubated overnight at 4°C with primary antibodies, washed with TBS-T (TBS with 0.1% Tween-20), and incubated with HRP-conjugated secondary antibodies at room temperature for 1 hour. Immuno-reactive protein was detected using SuperSignal West Pico Chem KIT (Thermo Scientific, USA). Primary antibodies used were against ERK, ERK pT202/pY204, Akt, Akt pS473, Src, Src pY416 (Cell Signaling), FAK (BD Transduction Laboratories), FAK pY397 (Millipore), α1(IV) (Abgent), α2(IV) (Abcam), DDR1, phosho-tyrosine (pY99), ubiquitin (Santa Cruz), α5(IV) (Proteintech), MEK1 (Abmart) and Actin (Sigma-Aldrich). Western blots were scanned and analyzed with Image J.

### Immunohistochemistry and immunofluorescence staining

Immunohistochemistry on 5-μm paraffin sections using antibodies against Ki-67 (Novocastra Laboratories), cleaved caspase-3 (Cell Signaling), CD31 (Abcam), α1(IV) (Abgent) or DDR1 (Santa Cruz) was performed as described [40]. For α5(IV) immuno-staining, 8-μm frozen tissue sections were fixed in cold acetone for 10 min. Samples were incubated with α5(IV) antibody (rat monoclonal antibody clone b14) (1:50–1:100) for 16 hours at 4°C, followed by incubation with Alexa Fluor 555/488 conjugated anti-rat IgG antibody. Immunohistochemistry or immunofluorescence sections were viewed under microscope (IX71; OLYMPUS, Inc.) with a UPlan-FLN 4×objective/0.13 PhL, a UPlan-FLN 10×objective/0.30 PhL, or a LUCPlan-FLN 20×objective/0.45 PhL. Images were captured with a digital camera (IX-SPT; OLYMPUS, Inc.) and Digital Acquire software (DPController; OLYMPUS, Inc.). Perfused blood vessels in Matrigel plugs were viewed by UV-illumination under microscope (SZX16; OLYMPUS, Inc.) with a SDF-PLAPO 1×PF. Images were captured with a digital camera (U-LH100HGAPO; OLYMPUS, Inc.) and Digital Acquire software (DPController; OLYMPUS, Inc.).

### Mouse treatment

All mice were housed in specific pathogen-free environment at the Shanghai Institute of Biochemistry and Cell Biology and treated in strict accordance with protocols approved by the Institutional Animal Care and Use Committee. *Col4a5* ^*LacZ*/Y^ mice were generated and maintained in C57/Bl background by the European Conditional Mouse Mutagenesis Program [13]. *Kras* ^*G12D*^ mice were back crossed to C57/Bl background 3 generations before cross with *Col4a5* ^*LacZ*/Y^ mice. LLC cells were transplanted at the armpit of lower limb of 8-week old WT or *Col4a5* ^*LacZ*/Y^ mice in C57/Bl background. To minimize the possible effects of mouse genetic background on tumor behavior, wild-type littermates were used as control for *Col4a5* ^*LacZ*/Y^ mice in all experiments. A549 cells were subcutaneously injected into Balb/c nude mice.

### Transmission electron microscopy (TEM)

Lung tissues isolated from 6-month old *Col4a5* ^+/Y^ and *Col4a5* ^*LacZ*/Y^ mice were fixed in 3% glutaraldehyde in 0.1M PBS (pH7.4) for 4 hours at room temperature and then in 1% osmium tetroxide overnight at 4°C. The fixed lung tissue were dehydrated through an alcohol series and embedded in Epon812 Resin at 60°C for 48 hours. Ultrathin sections (70 nm) were collected on copper grids. The grids were stained in 2% uranyl acetate for 40 minutes and in 0.5% lead citrate for 8 minutes orderly. The samples were examined under FEI Tecnai G2 Spirit TEM.

### Statistical analysis

Data were analyzed using the two-sided Student *t* test, and considered statistically significant when the *P* value was less than 0.05.

## Supporting Information

S1 Fig Generation of *Col4a5* knockout mice. (A) Structure of the targeting vector and *Col4a5* locus before and after homologous recombination. (B) Genotyping of *Col4a5* knockout mice. (C and D) RT-PCR analyses of total RNA from mouse embryonic fibroblasts (C) or lungs (D) detected a 263-bp amplimer corresponding to wild-type *Col4a5* RNA in wild-type (*Col4a5* ^+/Y^) samples that was absent in knockout (*Col4a5* ^*LacZ*/Y^) samples. Amplification of an *Actin* product was used as loading control. (E) Whole mount *LacZ* staining of lungs from *Col4a5* ^+/Y^ and *Col4a5* ^*LacZ*/Y^ mice. (F) Immunofluorescent staining shows α5(IV) chain is expressed in the lung bronchia and alveolar epithelial cells. Scale bars: 200μm.
(TIF)

S2 Fig Murine α5(IV) rescued the defects of α5(IV)-knockdown A549 cells. (A) Murine α5(IV) was expressed in α5(IV)-knockdown A549 cells. RT-PCR analyses of α5(IV) expression. (B-D) Expression of mouse α5(IV) in α5(IV)-knockdown A549 cells rescued the defects in cell proliferation (B), migration (C) and anchorage-independent growth (D). Data are presented as mean ± SD. ***P < 0.001. (E) Expression of mouse α5(IV) in α5(IV)-knockdown A549 cells restored DDR1 expression and ERK phosphorylation.
(TIF)

S3 Fig α5(IV) supports CRL-5810 lung cancer cell proliferation. (A) α5(IV) was knocked down in CRL-5810 lung cancer cells. Western blot analyses of phosphorylation levels of ERK, Akt, FAK and Src and α5(IV) expression in α5(IV)-knockdown CRL-5810 cells. (B and C) α5(IV) knockdown in CRL-5810 cells impaired cell proliferation (B) and anchorage-independent growth (C). Data are presented as mean ± SD. ***P < 0.001.
(TIF)

S4 Fig α5(IV) deficiency does not result in concomitant loss of α1(IV). (A) Eletron microscopy on lung sections from 6-month old *Col4a5* ^+/Y^ and *Col4a5* ^*LacZ*/Y^ mice. (B) α1(IV) staining on lung sections from 8-week and 6-month old *Col4a5* ^+/Y^ and *Col4a5* ^*LacZ*/Y^ mice. (C) α1(IV) staining on lung tumor sections from Kras/α5 WT and Kras/α5 KO mice. Scale bars: 200μm.
(TIF)

S5 Fig α1(IV) knockdown impaired lung cancer cell functions. Knockdown of α1(IV) in A549 cells impaired cell proliferation (A), cell migration (B) and anchorage-independent cell growth (C). Data are presented as mean ± SD. ***P < 0.001.
(TIF)

S6 Fig α1(IV) knockdown impaired endothelial cell functions. Knockdown of α1(IV) in HMEC-1 cells impaired cell proliferation (A), cell migration (B) and in vitro tubulogenesis (C). Data are presented as mean ± SD. *P < 0.05, ***P < 0.001. Scale bar: 200μm.
(TIF)

S7 Fig α5(IV) deficiency results in impaired activation of ERK in endothelial cells. (A) Western blot analyses of phosphorylation levels of ERK, Akt, FAK and Src and α1(IV), α2(IV) and α5(IV) expression in α5(IV)- or α1(IV)-knockdown HMEC-1 cells. (B) Western blot analyses of phosphorylation levels of ERK in α5(IV)-knockdown HMEC-1 cells expressing constitutively active MEK1. (C-E) Expression of constitutively active MEK1 in α5(IV)-knockdown HMEC-1 cells rescued the defects in cell proliferation (C), migration (D), and in vitro tubulogenesis (E). Data are presented as mean ± SD. **P < 0.01. Scale bar: 200 μm.
(TIF)

S8 Fig FACS analyses of integrin cell surface expression in A549 cells with α5(IV) knockdown. (TIF)

S9 Fig DDR1 is required for ERK activation, cell proliferation, migration and tubule formation in endothelial cells. (A) Western blot analyses of DDR1 expression in α5(IV)- or α1(IV)-knockdown HMEC-1 cells. (B) Western blot analyses of phosphorylation levels of ERK, Akt, FAK and Src in HMEC-1 cells with DDR1 knockdown. (C-E) Knockdown of DDR1 in HMEC-1 cells significantly impaired cell proliferation (C), migration (D) and in vitro tubulogenesis (E). Data are presented as mean ± SD. ***P < 0.001. Scale bar: 200 μm.
(TIF)

S10 Fig Overexpression of wild-type DDR1 is not sufficient to restore intracellular signaling in α5(IV)-knockdown A549 cells. Western blot analyses of phosphorylation levels of ERK and Akt in α5(IV)-knockdown A549 cells overexpressing wild-type DDR1.
(TIF)

S11 Fig Constitutively active DDR1 rescued the defects of α5(IV)-deficient endothelial cells. (A) Western blot analyses of DDR1 expression and phosphorylation levels of ERK, Akt, FAK and Src in α5(IV)-knockdown HMEC-1 cells expressing Div-DDR1 chimeric proteins. (B-D) Expression of Div-DDR1, but not mDiv-DDR1 or Div-DDR1 K655A in α5(IV)-knockdown HMEC-1 cells rescued the defects in cell proliferation (B), migration (C) and in vitro tubulogenesis (D). Data are presented as mean ± SD. ***P < 0.001. NS: not significant. Scale bar: 200 μm.
(TIF)

## References

[1] Kalluri R (2003) Basement membranes: structure, assembly and role in tumour angiogenesis. Nat Rev Cancer 3: 422–433. 12778132 10.1038/nrc1094

[2] Rowe RG, Weiss SJ (2008) Breaching the basement membrane: who, when and how? Trends Cell Biol 18: 560–574. 10.1016/j.tcb.2008.08.007 18848450

[3] Van Agtmael T, Bruckner-Tuderman L (2010) Basement membranes and human disease. Cell Tissue Res 339: 167–188. 10.1007/s00441-009-0866-y 19756754

[4] Hudson BG, Tryggvason K, Sundaramoorthy M, Neilson EG (2003) Alport's syndrome, Goodpasture's syndrome, and type IV collagen. N Engl J Med 348: 2543–2556. 12815141 10.1056/NEJMra022296

[5] Khoshnoodi J, Pedchenko V, Hudson BG (2008) Mammalian collagen IV. Microsc Res Tech 71: 357–370. 10.1002/jemt.20564 18219669 PMC4788096

[6] Burnier JV, Wang N, Michel RP, Hassanain M, Li S, et al (2011) Type IV collagen-initiated signals provide survival and growth cues required for liver metastasis. Oncogene 30: 3766–3783. 10.1038/onc.2011.89 21478904

[7] Noel A, De Pauw-Gillet MC, Purnell G, Nusgens B, Lapiere CM, et al (1993) Enhancement of tumorigenicity of human breast adenocarcinoma cells in nude mice by matrigel and fibroblasts. Br J Cancer 68: 909–915. 8217606 10.1038/bjc.1993.453 PMC1968733

[8] Sethi T, Rintoul RC, Moore SM, MacKinnon AC, Salter D, et al (1999) Extracellular matrix proteins protect small cell lung cancer cells against apoptosis: a mechanism for small cell lung cancer growth and drug resistance in vivo. Nat Med 5: 662–668. 10371505 10.1038/9511

[9] Senger DR, Davis GE (2011) Angiogenesis. Cold Spring Harb Perspect Biol 3: a005090 10.1101/cshperspect.a005090 21807843 PMC3140681

[10] Sund M, Xie L, Kalluri R (2004) The contribution of vascular basement membranes and extracellular matrix to the mechanics of tumor angiogenesis. APMIS 112: 450–462. 15563309 10.1111/j.1600-0463.2004.t01-1-apm11207-0806.x

[11] Macias-Perez I, Borza C, Chen X, Yan X, Ibanez R, et al (2008) Loss of integrin alpha1beta1 ameliorates Kras-induced lung cancer. Cancer Res 68: 6127–6135. 10.1158/0008-5472.CAN-08-1395 18676835 PMC2801028

[12] Pozzi A, Moberg PE, Miles LA, Wagner S, Soloway P, et al (2000) Elevated matrix metalloprotease and angiostatin levels in integrin alpha 1 knockout mice cause reduced tumor vascularization. Proc Natl Acad Sci U S A 97: 2202–2207. 10681423 10.1073/pnas.040378497 PMC15778

[13] Skarnes WC, Rosen B, West AP, Koutsourakis M, Bushell W, et al (2011) A conditional knockout resource for the genome-wide study of mouse gene function. Nature 474: 337–342. 10.1038/nature10163 21677750 PMC3572410

[14] Kim SH, Turnbull J, Guimond S (2011) Extracellular matrix and cell signalling: the dynamic cooperation of integrin, proteoglycan and growth factor receptor. J Endocrinol 209: 139–151. 10.1530/JOE-10-0377 21307119

[15] Campbell PM, Groehler AL, Lee KM, Ouellette MM, Khazak V, et al (2007) K-Ras promotes growth transformation and invasion of immortalized human pancreatic cells by Raf and phosphatidylinositol 3-kinase signaling. Cancer Res 67: 2098–2106. 17332339 10.1158/0008-5472.CAN-06-3752

[16] Van Meter ME, Diaz-Flores E, Archard JA, Passegue E, Irish JM, et al (2007) K-RasG12D expression induces hyperproliferation and aberrant signaling in primary hematopoietic stem/progenitor cells. Blood 109: 3945–3952. 17192389 10.1182/blood-2006-09-047530 PMC1874575

[17] Rigden MD, Baier C, Ramirez-Arcos S, Liao M, Wang M, et al (2008) Identification of the coiled-coil domains of Enterococcus faecalis DivIVA that mediate oligomerization and their importance for biological function. J Biochem 144: 63–76. 10.1093/jb/mvn044 18388125

[18] Zhang Y, Mao F, Lu Y, Wu W, Zhang L, et al (2011) Transduction of the Hedgehog signal through the dimerization of Fused and the nuclear translocation of Cubitus interruptus. Cell Res 21: 1436–1451. 10.1038/cr.2011.136 21844892 PMC3193457

[19] Olaso E, Ikeda K, Eng FJ, Xu L, Wang LH, et al (2001) DDR2 receptor promotes MMP-2-mediated proliferation and invasion by hepatic stellate cells. J Clin Invest 108: 1369–1378. 11696582 10.1172/JCI12373 PMC209436

[20] Vogel W, Brakebusch C, Fassler R, Alves F, Ruggiero F, et al (2000) Discoidin domain receptor 1 is activated independently of beta(1) integrin. J Biol Chem 275: 5779–5784. 10681566 10.1074/jbc.275.8.5779

[21] Carafoli F, Hohenester E (2013) Collagen recognition and transmembrane signalling by discoidin domain receptors. Biochim Biophys Acta 1834: 2187–2194. 10.1016/j.bbapap.2012.10.014 23128141 PMC4332414

[22] Leitinger B, Hohenester E (2007) Mammalian collagen receptors. Matrix Biol 26: 146–155. 17141492 10.1016/j.matbio.2006.10.007

[23] Rikova K, Guo A, Zeng Q, Possemato A, Yu J, et al (2007) Global survey of phosphotyrosine signaling identifies oncogenic kinases in lung cancer. Cell 131: 1190–1203. 18083107 10.1016/j.cell.2007.11.025

[24] Ford CE, Lau SK, Zhu CQ, Andersson T, Tsao MS, et al (2007) Expression and mutation analysis of the discoidin domain receptors 1 and 2 in non-small cell lung carcinoma. Br J Cancer 96: 808–814. 17299390 10.1038/sj.bjc.6603614 PMC2360060

[25] Valencia K, Ormazabal C, Zandueta C, Luis-Ravelo D, Anton I, et al (2012) Inhibition of collagen receptor discoidin domain receptor-1 (DDR1) reduces cell survival, homing, and colonization in lung cancer bone metastasis. Clin Cancer Res 18: 969–980. 10.1158/1078-0432.CCR-11-1686 22223527

[26] Yeh YC, Lin HH, Tang MJ (2012) A tale of two collagen receptors, integrin beta1 and discoidin domain receptor 1, in epithelial cell differentiation. Am J Physiol Cell Physiol. 10.1152/ajpcell.00253.2012 23015544

[27] Shintani Y, Fukumoto Y, Chaika N, Svoboda R, Wheelock MJ, et al (2008) Collagen I-mediated up-regulation of N-cadherin requires cooperative signals from integrins and discoidin domain receptor 1. J Cell Biol 180: 1277–1289. 10.1083/jcb.200708137 18362184 PMC2290851

[28] Gould DB, Phalan FC, Breedveld GJ, van Mil SE, Smith RS, et al (2005) Mutations in Col4a1 cause perinatal cerebral hemorrhage and porencephaly. Science 308: 1167–1171. 15905400 10.1126/science.1109418

[29] Poschl E, Schlotzer-Schrehardt U, Brachvogel B, Saito K, Ninomiya Y, et al (2004) Collagen IV is essential for basement membrane stability but dispensable for initiation of its assembly during early development. Development 131: 1619–1628. 14998921 10.1242/dev.01037

[30] Cosgrove D, Meehan DT, Grunkemeyer JA, Kornak JM, Sayers R, et al (1996) Collagen COL4A3 knockout: a mouse model for autosomal Alport syndrome. Genes Dev 10: 2981–2992. 8956999 10.1101/gad.10.23.2981

[31] Miner JH, Sanes JR (1996) Molecular and functional defects in kidneys of mice lacking collagen alpha 3(IV): implications for Alport syndrome. J Cell Biol 135: 1403–1413. 8947561 10.1083/jcb.135.5.1403 PMC2121079

[32] Rheault MN, Kren SM, Thielen BK, Mesa HA, Crosson JT, et al (2004) Mouse model of X-linked Alport syndrome. J Am Soc Nephrol 15: 1466–1474. 15153557 10.1097/01.asn.0000130562.90255.8f

[33] Kalluri R, Shield CF, Todd P, Hudson BG, Neilson EG (1997) Isoform switching of type IV collagen is developmentally arrested in X-linked Alport syndrome leading to increased susceptibility of renal basement membranes to endoproteolysis. J Clin Invest 99: 2470–2478. 9153291 10.1172/JCI119431 PMC508088

[34] Ghajar CM, Peinado H, Mori H, Matei IR, Evason KJ, et al (2013) The perivascular niche regulates breast tumour dormancy. Nat Cell Biol. 10.1038/ncb2767 PMC3826912 23728425

[35] Ingber D, Folkman J (1988) Inhibition of angiogenesis through modulation of collagen metabolism. Lab Invest 59: 44–51. 2455830

[36] Kohda T, Okada S, Hayashi A, Kanzaki S, Ninomiya Y, et al (2004) High nephritogenicity of monoclonal antibodies belonging to IgG2a and IgG2b subclasses in rat anti-GBM nephritis. Kidney Int 66: 177–186. 15200424 10.1111/j.1523-1755.2004.00719.x

[37] Chen J, Yang W, Kim M, Carman CV, Springer TA (2006) Regulation of outside-in signaling and affinity by the beta2 I domain of integrin alphaLbeta2. Proc Natl Acad Sci U S A 103: 13062–13067. 16920795 10.1073/pnas.0605666103 PMC1559753

[38] Naldini L, Blomer U, Gage FH, Trono D, Verma IM (1996) Efficient transfer, integration, and sustained long-term expression of the transgene in adult rat brains injected with a lentiviral vector. Proc Natl Acad Sci U S A 93: 11382–11388. 8876144 10.1073/pnas.93.21.11382 PMC38066

[39] Mansour SJ, Matten WT, Hermann AS, Candia JM, Rong S, et al (1994) Transformation of mammalian cells by constitutively active MAP kinase kinase. Science 265: 966–970. 8052857 10.1126/science.8052857

[40] Gao Y, Xiao Q, Ma H, Li L, Liu J, et al (2010) LKB1 inhibits lung cancer progression through lysyl oxidase and extracellular matrix remodeling. Proc Natl Acad Sci U S A 107: 18892–18897. 10.1073/pnas.1004952107 20956321 PMC2973865

[41] Ge G, Hopkins DR, Ho WB, Greenspan DS (2005) GDF11 forms a bone morphogenetic protein 1-activated latent complex that can modulate nerve growth factor-induced differentiation of PC12 cells. Mol Cell Biol 25: 5846–5858. 15988002 10.1128/MCB.25.14.5846-5858.2005 PMC1168807

[42] Montesano R, Orci L, Vassalli P (1983) In vitro rapid organization of endothelial cells into capillary-like networks is promoted by collagen matrices. J Cell Biol 97: 1648–1652. 6630296 10.1083/jcb.97.5.1648 PMC2112683

[43] Akhtar N, Dickerson EB, Auerbach R (2002) The sponge/Matrigel angiogenesis assay. Angiogenesis 5: 75–80. 12549862 10.1023/a:1021507031486

[44] Poon RT, Ng IO, Lau C, Yu WC, Yang ZF, et al (2002) Tumor microvessel density as a predictor of recurrence after resection of hepatocellular carcinoma: a prospective study. J Clin Oncol 20: 1775–1785. 11919234 10.1200/JCO.2002.07.089

