# Supplementary figures and images for: Minor Type IV Collagen α5 Chain Promotes Cancer Progression through Discoidin Domain Receptor-1
Source: PLoS Genet. 2015 May 19;11(5):e1005249. doi: 10.1371/journal.pgen.1005249 (PMC4438069; doi:10.1371/journal.pgen.1005249)

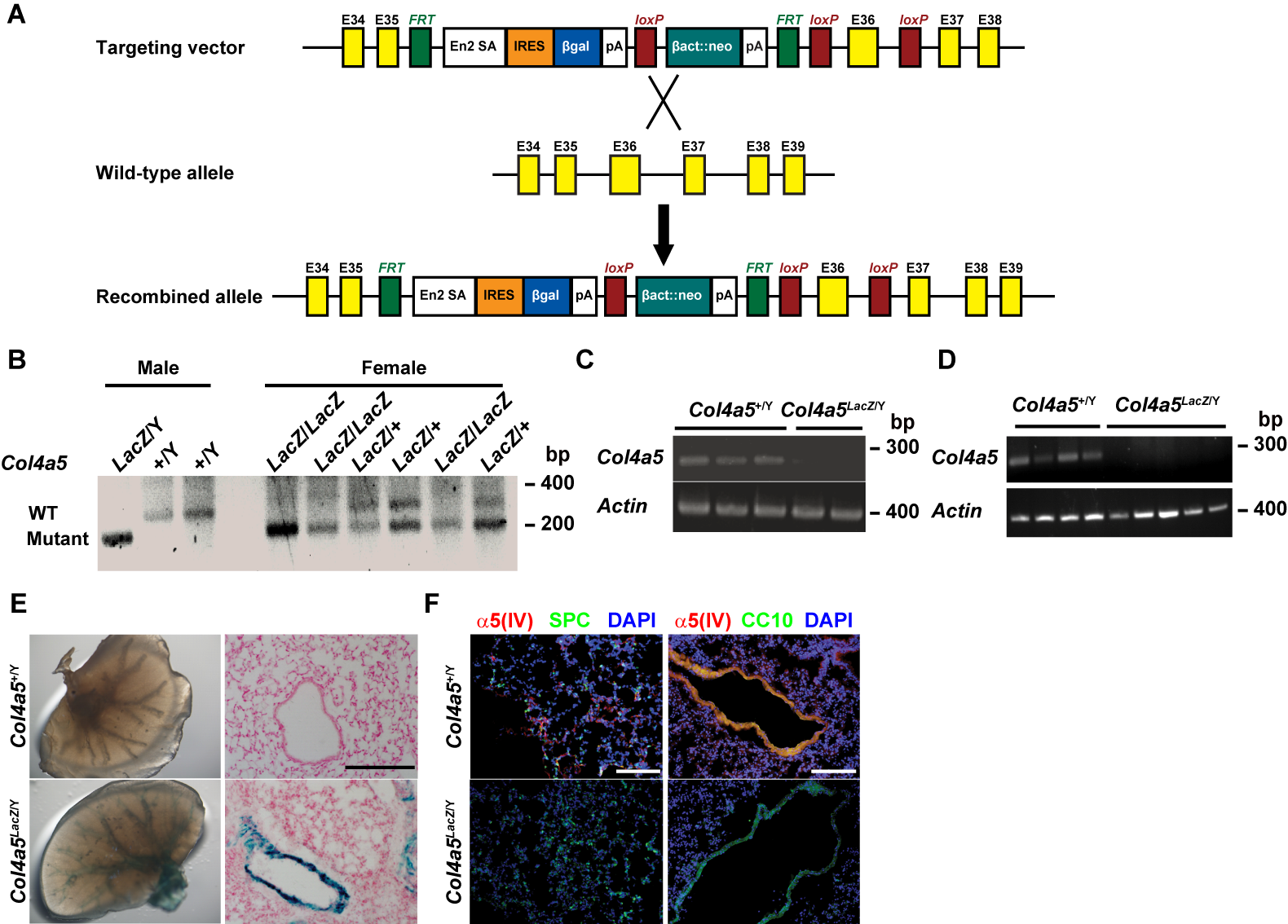

Supplement: S1 Fig — (A) Structure of the targeting vector and Col4a5 locus before and after homologous recombination. (B) Genotyping of Col4a5 knockout mice. (C and D) RT-PCR analyses of total RNA from mouse embryonic fibroblasts (C) or lungs (D) detected a 263-bp amplimer corresponding to wild-type Col4a5 RNA in wild-type (Col4a5 +/Y) samples that was absent in knockout (Col4a5 LacZ/Y) samples. Amplification of an Actin product was used as loading control. (E) Whole mount LacZ staining of lungs from Col4a5 +/Y and Col4a5 LacZ/Y mice. (F) Immunofluorescent staining shows α5(IV) chain is expressed in the lung bronchia and alveolar epithelial cells. Scale bars: 200μm. (TIF) [file pgen.1005249.s001.tif]

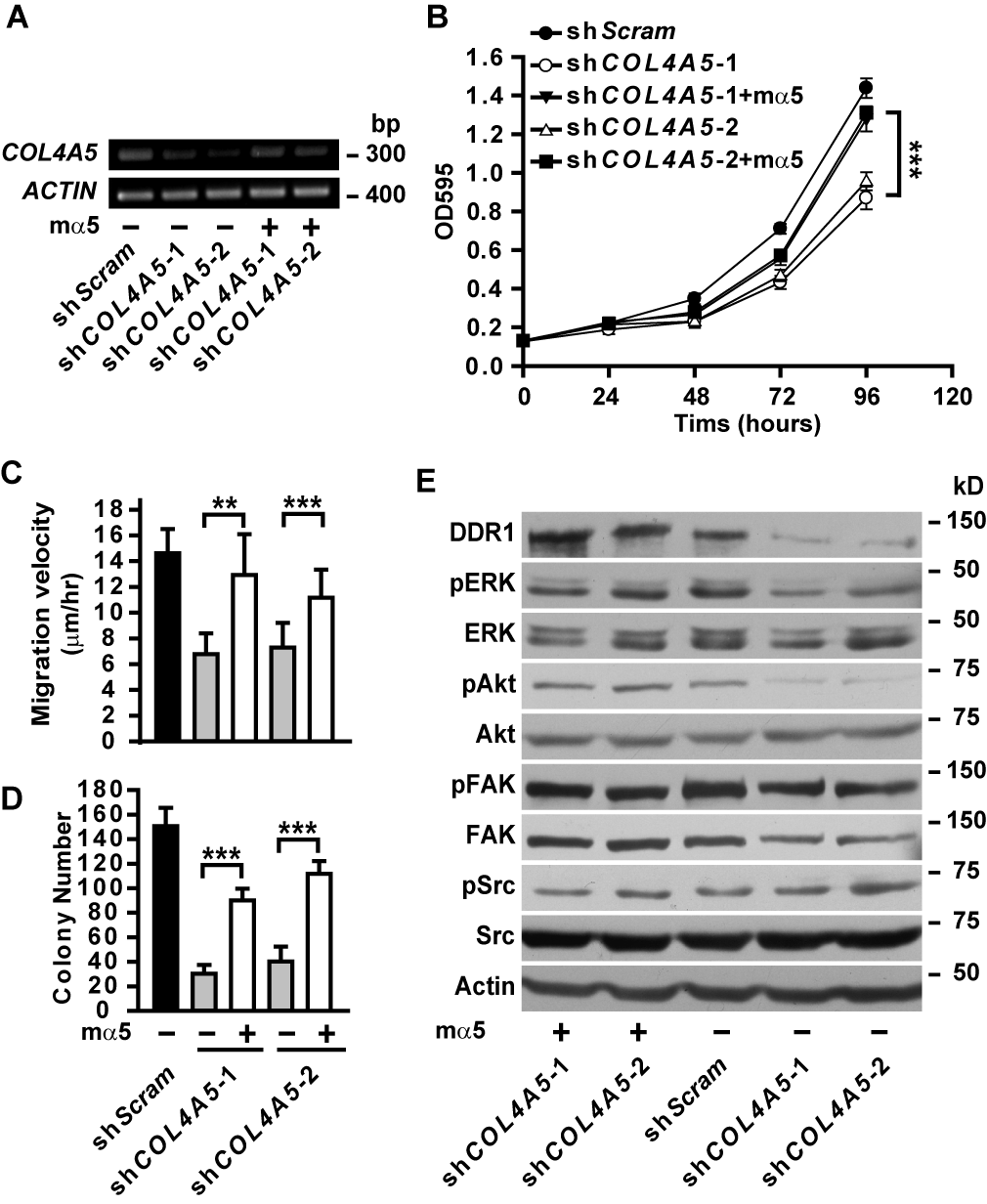

Supplement: S2 Fig — (A) Murine α5(IV) was expressed in α5(IV)-knockdown A549 cells. RT-PCR analyses of α5(IV) expression. (B-D) Expression of mouse α5(IV) in α5(IV)-knockdown A549 cells rescued the defects in cell proliferation (B), migration (C) and anchorage-independent growth (D). Data are presented as mean ± SD. ***P < 0.001. (E) Expression of mouse α5(IV) in α5(IV)-knockdown A549 cells restored DDR1 expression and ERK phosphorylation. (TIF) [file pgen.1005249.s002.tif]

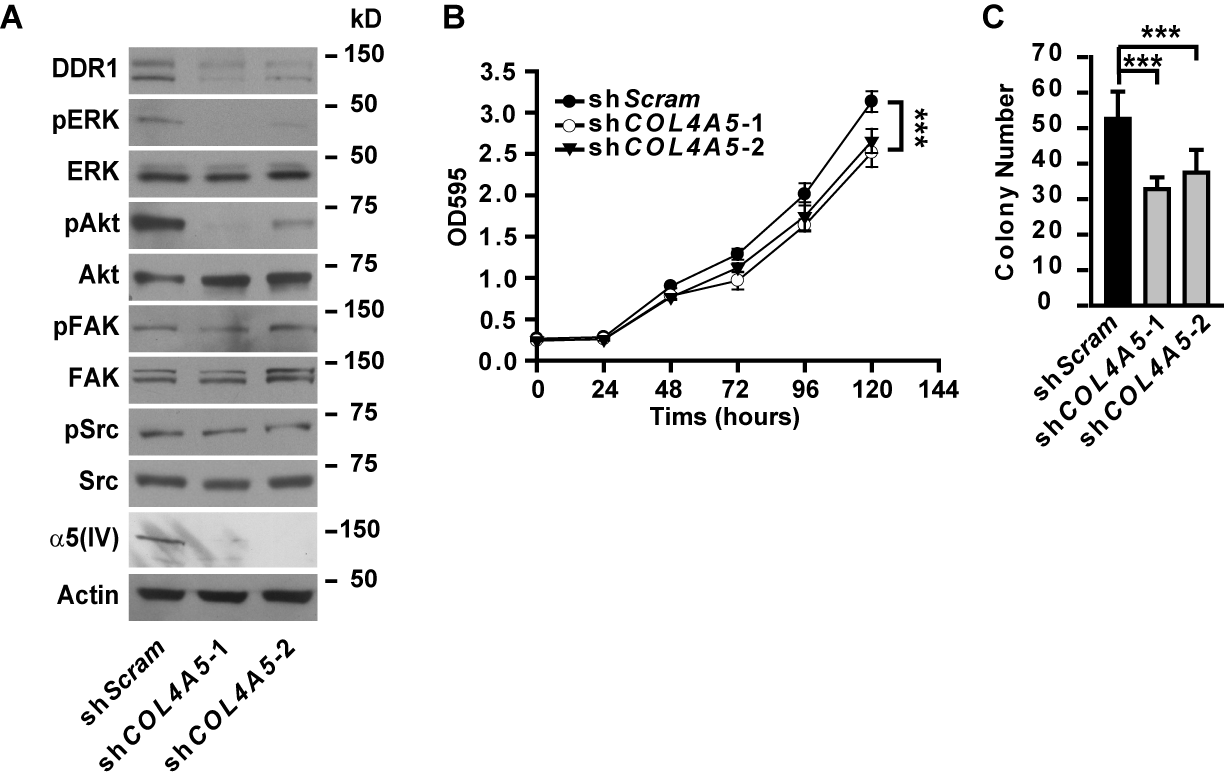

Supplement: S3 Fig — (A) α5(IV) was knocked down in CRL-5810 lung cancer cells. Western blot analyses of phosphorylation levels of ERK, Akt, FAK and Src and α5(IV) expression in α5(IV)-knockdown CRL-5810 cells. (B and C) α5(IV) knockdown in CRL-5810 cells impaired cell proliferation (B) and anchorage-independent growth (C). Data are presented as mean ± SD. ***P < 0.001. (TIF) [file pgen.1005249.s003.tif]

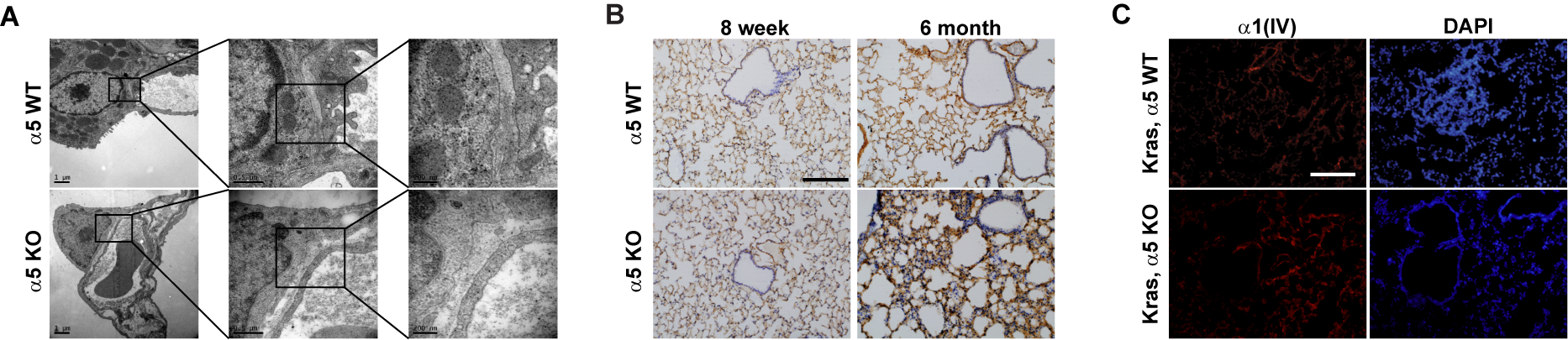

Supplement: S4 Fig — (A) Eletron microscopy on lung sections from 6-month old Col4a5 +/Y and Col4a5 LacZ/Y mice. (B) α1(IV) staining on lung sections from 8-week and 6-month old Col4a5 +/Y and Col4a5 LacZ/Y mice. (C) α1(IV) staining on lung tumor sections from Kras/α5 WT and Kras/α5 KO mice. Scale bars: 200μm. (TIF) [file pgen.1005249.s004.tif]

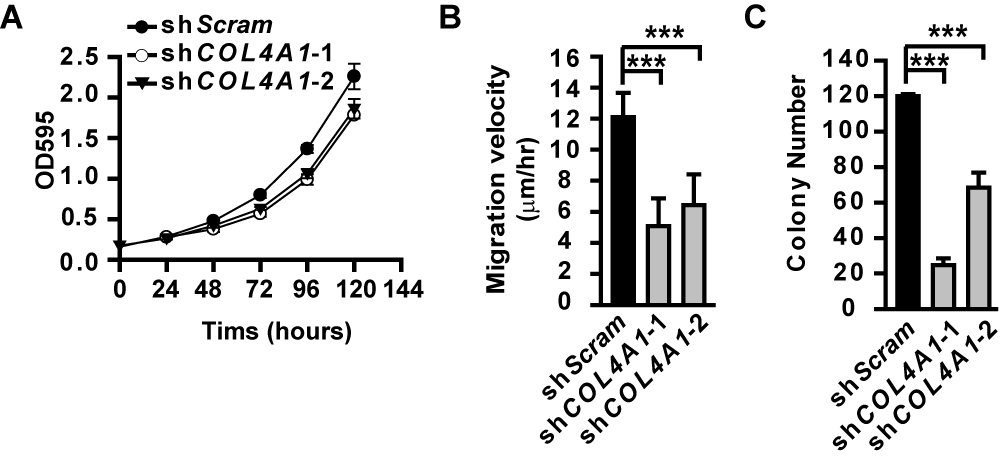

Supplement: S5 Fig — Knockdown of α1(IV) in A549 cells impaired cell proliferation (A), cell migration (B) and anchorage-independent cell growth (C). Data are presented as mean ± SD. ***P < 0.001. (TIF) [file pgen.1005249.s005.tif]

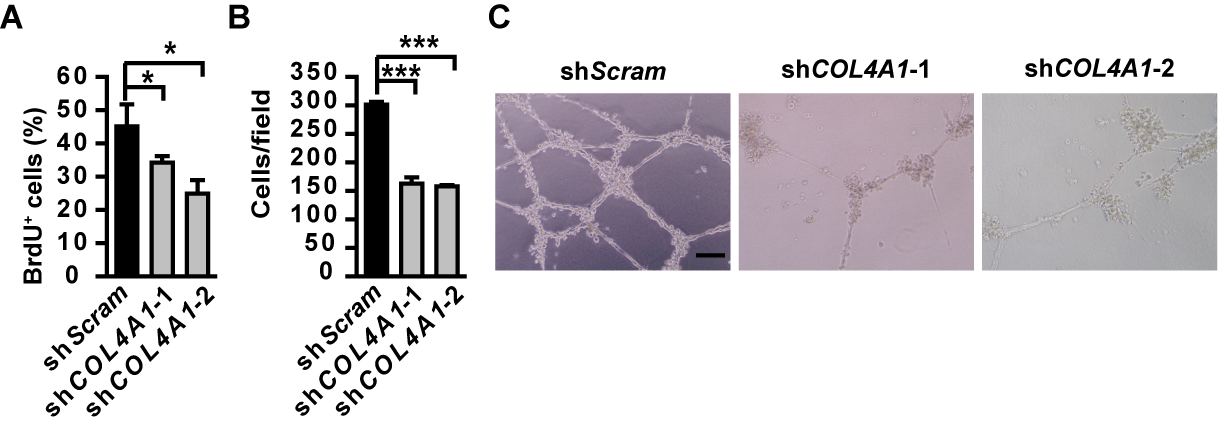

Supplement: S6 Fig — Knockdown of α1(IV) in HMEC-1 cells impaired cell proliferation (A), cell migration (B) and in vitro tubulogenesis (C). Data are presented as mean ± SD. *P < 0.05, ***P < 0.001. Scale bar: 200μm. (TIF) [file pgen.1005249.s006.tif]

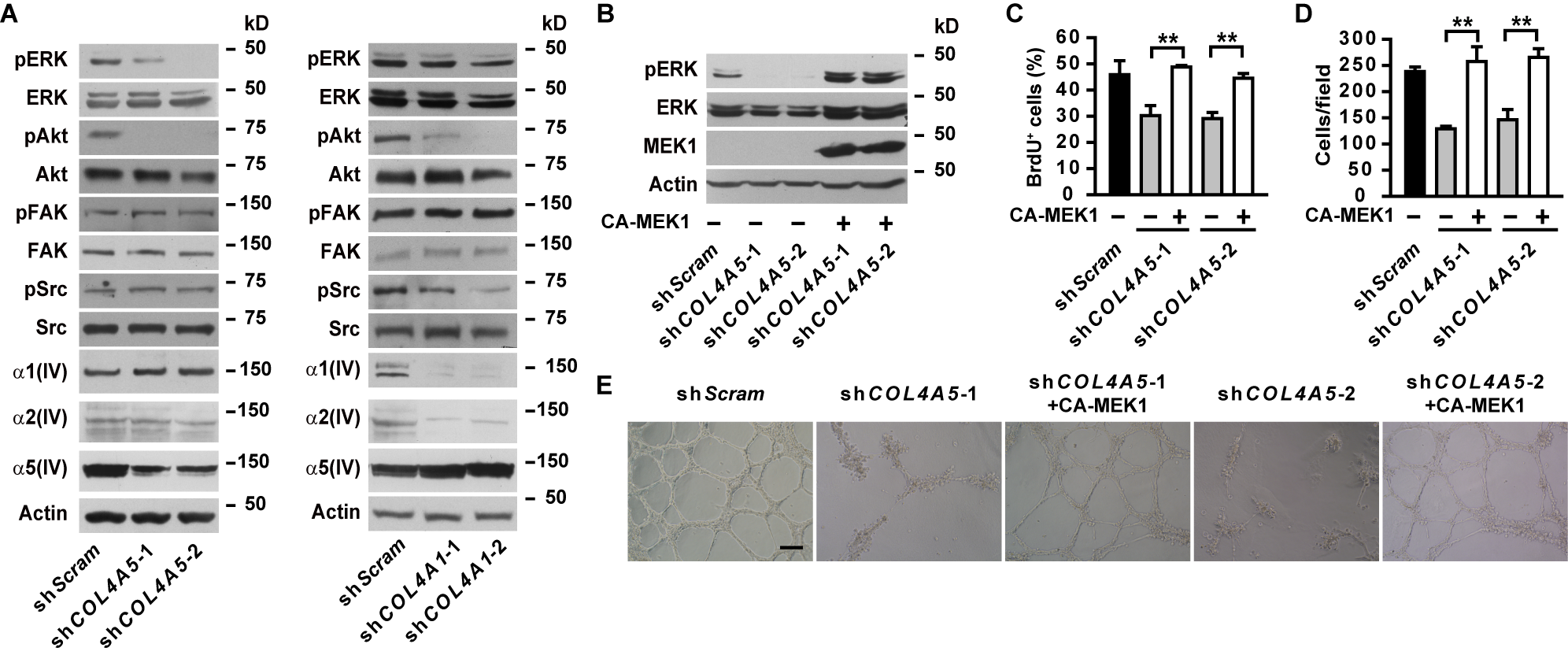

Supplement: S7 Fig — (A) Western blot analyses of phosphorylation levels of ERK, Akt, FAK and Src and α1(IV), α2(IV) and α5(IV) expression in α5(IV)- or α1(IV)-knockdown HMEC-1 cells. (B) Western blot analyses of phosphorylation levels of ERK in α5(IV)-knockdown HMEC-1 cells expressing constitutively active MEK1. (C-E) Expression of constitutively active MEK1 in α5(IV)-knockdown HMEC-1 cells rescued the defects in cell proliferation (C), migration (D), and in vitro tubulogenesis (E). Data are presented as mean ± SD. **P < 0.01. Scale bar: 200 μm. (TIF) [file pgen.1005249.s007.tif]

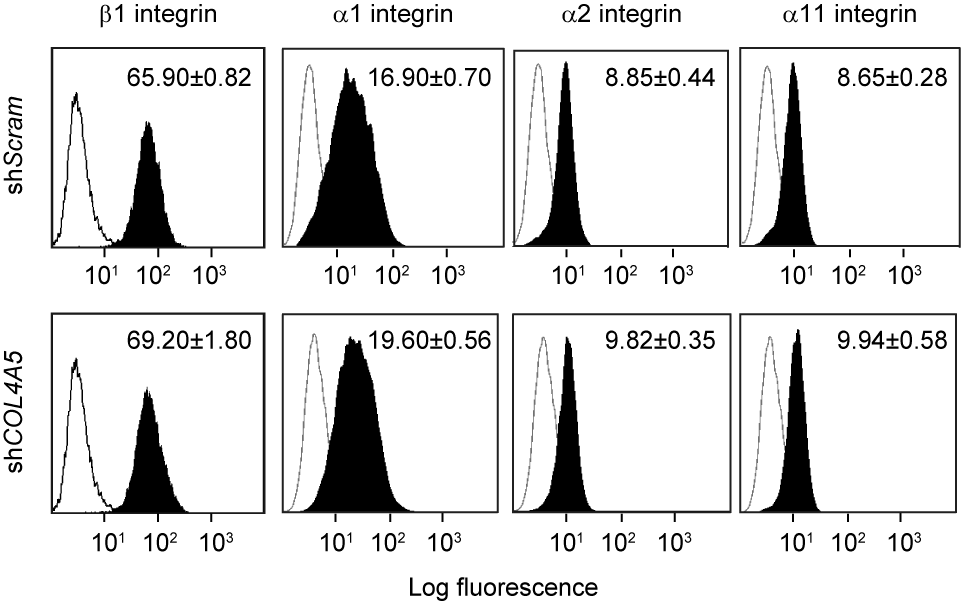

Supplement: S8 Fig — (TIF) [file pgen.1005249.s008.tif]

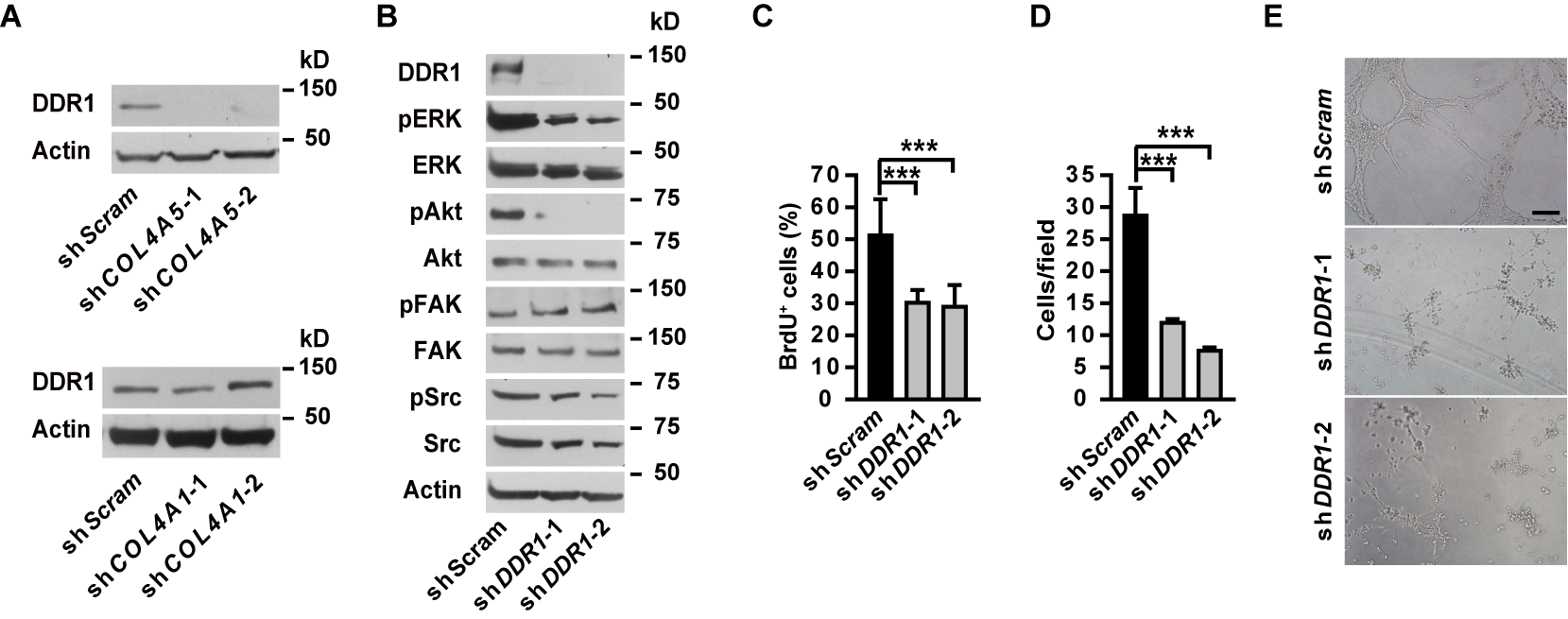

Supplement: S9 Fig — (A) Western blot analyses of DDR1 expression in α5(IV)- or α1(IV)-knockdown HMEC-1 cells. (B) Western blot analyses of phosphorylation levels of ERK, Akt, FAK and Src in HMEC-1 cells with DDR1 knockdown. (C-E) Knockdown of DDR1 in HMEC-1 cells significantly impaired cell proliferation (C), migration (D) and in vitro tubulogenesis (E). Data are presented as mean ± SD. ***P < 0.001. Scale bar: 200 μm. (TIF) [file pgen.1005249.s009.tif]

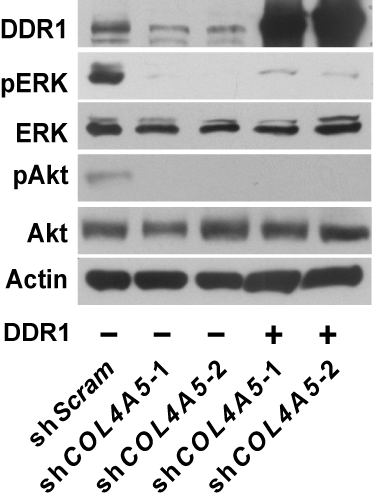

Supplement: S10 Fig — Western blot analyses of phosphorylation levels of ERK and Akt in α5(IV)-knockdown A549 cells overexpressing wild-type DDR1. (TIF) [file pgen.1005249.s010.tif]

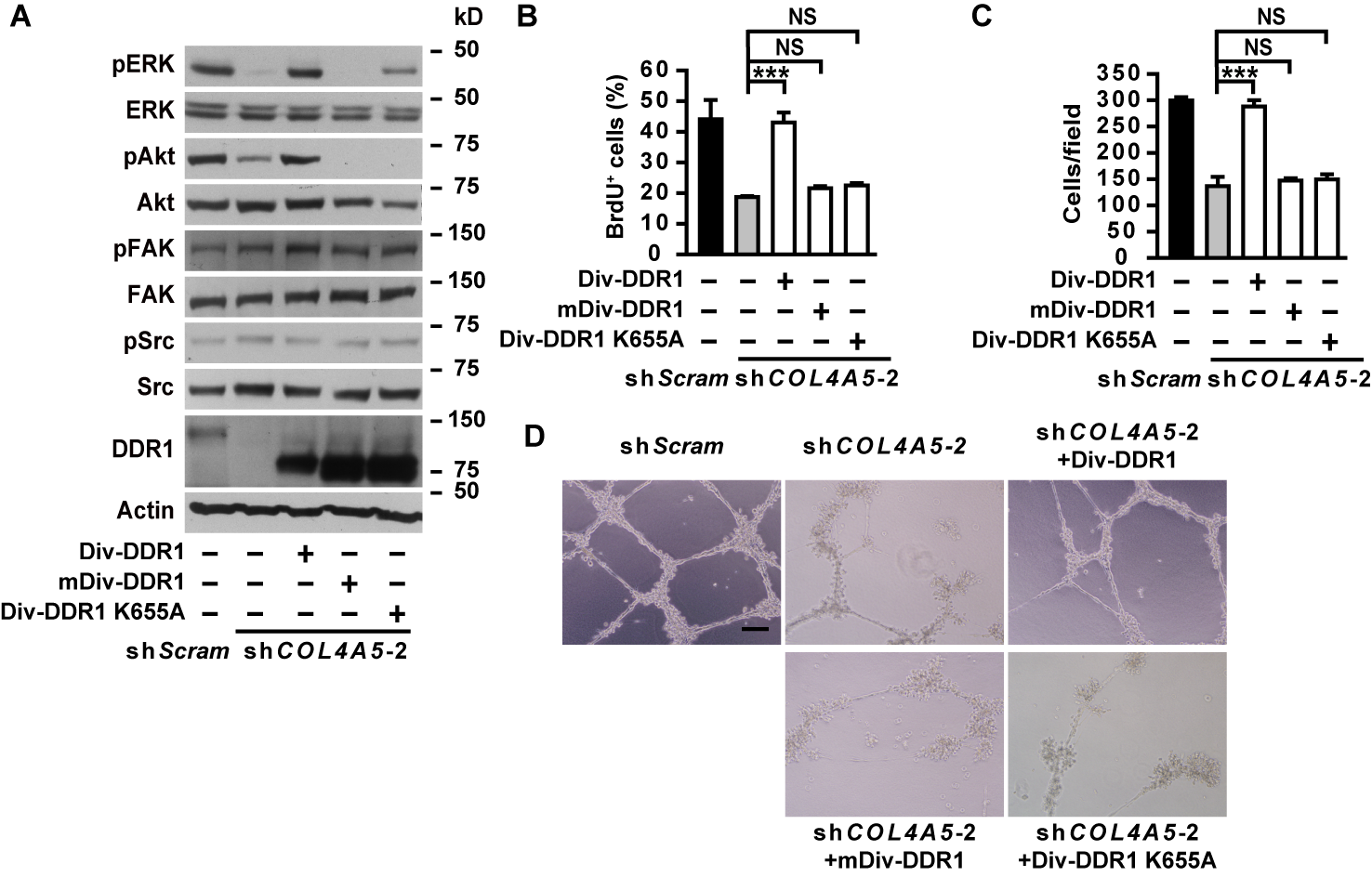

Supplement: S11 Fig — (A) Western blot analyses of DDR1 expression and phosphorylation levels of ERK, Akt, FAK and Src in α5(IV)-knockdown HMEC-1 cells expressing Div-DDR1 chimeric proteins. (B-D) Expression of Div-DDR1, but not mDiv-DDR1 or Div-DDR1 K655A in α5(IV)-knockdown HMEC-1 cells rescued the defects in cell proliferation (B), migration (C) and in vitro tubulogenesis (D). Data are presented as mean ± SD. ***P < 0.001. NS: not significant. Scale bar: 200 μm. (TIF) [file pgen.1005249.s011.tif]
